# Isolation of Secondary Metabolites from *Achillea grandifolia* Friv. (Asteraceae) and Main Compounds’ Effects on a Glioblastoma Cellular Model

**DOI:** 10.3390/pharmaceutics15051383

**Published:** 2023-04-30

**Authors:** Olga S. Tsiftsoglou, Nikos Krigas, Christos Gounaris, Christina Papitsa, Maria Nanouli, Evrysthenis Vartholomatos, Georgios S. Markopoulos, Rafaela Isyhou, George Alexiou, Diamanto Lazari

**Affiliations:** 1 Laboratory of Pharmacognosy, School of Pharmacy, Faculty of Health Sciences, Aristotle University of Thessaloniki, 54124 Thessaloniki, Greece; 2 Institute of Plant Breeding and Genetic Resources, Hellenic Agricultural Organization—Demeter, 57001 Thermi, Greece; 3 Neurosurgical Institute, University of Ioannina School of Medicine, 45110 Ioannina, Greece; 4 Haematology Laboratory—Unit of Molecular Biology and Translational Flow Cytometry, University Hospital of Ioannina, 45500 Ioannina, Greece; 5 Department of Neurosurgery, University Hospital of Ioannina, 45500 Ioannina, Greece

**Keywords:** flavonoids, glioblastoma, rupicolin A, rupicolin B, sesquiterpene lactones, cell lines

## Abstract

This study aims at the isolation and structural determination of the secondary metabolites of the herbaceous perennial plant *Achillea grandifolia* Friv. (Asteraceae). The examination of the non-volatile content of the leaves and flowers of *A. grandifolia* afforded the isolation of sixteen secondary metabolites. On the basis of NMR spectra, the identified compounds included ten sesquiterpene lactones; three guaianolides—rupicolin A (**1**), rupicolin B (**2**), and (4S,6aS,9R,9aS,9bS)-4,6a,9-trihydroxy-9-methyl-3,6-dimethylene-3a,4,5,6,6a,9,9a,9b-octahydro-3H-azuleno [4,5-b]furan-2-one (**3**); two eudesmanolides—artecalin (**4**) and ridentin B (**5**); two sesquiterpene methyl esters—(1S,2S,4αR,5R,8R,8αS)-decahydro-1,5,8-trihydroxy-4α,8-dimethyl–methylene-2-naphthaleneacetic acid methylester (**6**) and 1β, 3β, 6α-trihydroxycostic acid methyl ester (**7**); three secoguaianolides—acrifolide (**8**), arteludovicinolide A (**9**), and lingustolide A (**10**); and an iridoid—loliolide (**11**). Moreover, five known flavonoids, i.e., apigenin, luteolin, eupatolitin, apigenin 7-*O*-glucoside, and luteolin 7-O-glucoside (**12-16**) were also purified from the aerial parts of the plant material. We also investigated the effect of rupicolin A (**1**) and B (**2**) (main compounds) on U87MG and T98G glioblastoma cell lines. An MTT assay was performed to define cytotoxic effects and to calculate the IC50, while flow cytometry was employed to analyze the cell cycle. The IC50 values of reduced viability during the 48 h treatment for compound (**1**) and (**2**) were 38 μM and 64 μM for the U87MG cells and 15 μM and 26 μM for the T98G cells, respectively. Both rupicolin A and B induced a G2/M cell cycle arrest.

## 1. Introduction

The genus *Achillea* has a great pharmacological importance and belongs to the Asteraceae family; this genus includes more than 130 wild-growing species worldwide, which are mainly distributed in the northern hemisphere, and almost 40% occur in the Balkan peninsula [1]. Due to numerous medicinal properties (anti-microbial, antioxidant, anti-inflammatory, wound healing activity, anti-diabetic activity), the aerial parts of several members of this genus have been used extensively in folk medicine according to many ethnopharmacological studies [2,3]. Numerous phytochemical studies have reported that members of the genus *Achillea* are usually terpenoid- rich (mainly sesquiterpene lactones) and phenolic-rich plants with phenolic acids, flavonoids, and coumarins. Moreover, many investigations have proved the cytotoxic effects of natural products (extracts, essential oils, and isolated compounds) from different *Achillea* species such as *A. millefolium* L., *A. clavennae* L., *A. talagonica* Boiss., *A. wilhelmsii* C. Koch, *A. fragrantissima* (Forssk.) Sch. Bip., *A. teretifolia* Willd., and *A. coarctata* Poir. [4,5,6,7,8].

*Achillea grandifolia* Friv. is an herbaceous perennial plant, which is subendemic to the Balkan Peninsula with populations in adjacent Anatolia in Turkey. Previous studies have examined infusions of *A. grandifolia* with a focus on the antioxidant activity, total phenol, and total flavonoid contents of the extracts, as well as on its essential oil composition [9,10]. The methanol extract from *A. grandifolia* collected from the Balkan Peninsula is reported to have antibacterial and antioxidant properties [11]. The first research aiming to detect secondary metabolites in *A. grandifolia* [12] have led to the isolation of 6-hydroxyluteolin 6-methyl ether (nepetin), coumarin esculetin, and a flavonol that was assumed to be quercetin dimethyl ether; all were characterized using chromatographic technics (TLC) compared to standards and were coupled with mass and UV spectroscopy. However, no NMR spectra of these compounds have ever been presented [12]. In this line, another older study examining the roots of *A. grandifolia* have revealed the presence of two piperidides [13].

Several phytochemical investigations in other *Achillea* species indicate that many secondary metabolites isolated from members of this genus are highly bioactive [14]. For example, the anti-spasmodic flavonoids cynaroside, cosmosiin, 3β-methoxy-iso-seco-tanapartholide, tanaphillin have all been isolated from *A. millefolium;* iso-seco-tanapartholide and 8-hydroxy-3-methoxy-iso-seco-tanaparatholide have been isolated from *A. falcata* L. and are associated with the significant ability to inhibit HaCaT-cell growth by significantly decreasing the viability of keratinocyte cells [14]. Other examples refer to ligustolide-A and arteludovicinolide-A that exhibit anti-inflammatory properties or 1α,6α,8α-trihydroxy-5α,7β H-guaia-3,10,14,11,13-trien-12-oic acid and 1α,6α,8α-trihydroxy-5α,7βH-guaia-3,9,11,13-trien-12-oic acid, which may significantly enhance the proliferation of beneficial macrophages [14]. Moreover, in a recent study, the protective effect of rupicolin A and acrifolide has been investigated against iodixanol-induced cytotoxicity in cultured renal tubular cells (LLC-PK1), although none of them showed any toxic effect at 25μΜ and 50μΜ, respectively [15].

To date, there are no reports in the literature concerning the isolation of non-volatile terpenoid compounds from *A. grandifolia*; to fill the existing gap, we report herein the findings of the investigation performed separately on its flowers (compound head inflorescences) and compound leaves. Moreover, the potential anti-proliferative role of rupicoline A (**1**) and B (**2**) in cancer cells such as glioblastoma (GBM) has never been studied. Therefore, we investigated the effect of these two main compounds in two GBM cell lines as well.

## 2. Materials and Methods

### 2.1. General Experimental Procedures

Column chromatography (CC) was carried out on silica gel 60 (Merck Art. 9385, Darmstadt, Germany), gradient elution, with the solvent mixtures indicated in each case. Vacuum liquid chromatography (VLC) was carried out on silica gel 60 H (Merck Art. 7736), gradient elution, with the solvent mixtures indicated in each case. Thin liquid chromatography (TLC) was carried out on silica gel plates (Kieselgel F254, Merck, Art. 5554); detection on TLC plates: UV light (absorbance: 254 and 366 nm). For the visualization of the chromatograms on silica gel, vanillin–H_2_SO_4_ spray reagent was used. For the high-performance liquid chromatography (HPLC), a Lab Alliance Series III pump equipped with Clarity software and a Shodex RI Detector was used using a C18 ODS1 Spherisorb with a 10μm column that measured 250 mm × 10 mm (Waters).

Spectroscopic NMR data: The ^1^H-NMR and ^13^C-NMR spectra were recorded in CD_3_OD using AGILENT DD2 500 (500.1 MHz for ^1^H-NMR and 125.5 MHz for ^13^C-NMR) spectrometers. The chemical shifts are provided in δ (ppm) values relative to TMS (3.31 ppm for ^1^H-NMR and 49.05 ppm for ^13^C-NMR).

Plant material: The above-ground part of 10–15 wild-growing plants was collected in June 2019 from Mt. Pelion (Southeastern Thessaly, Greece; 39°26′18.84″ N, 23°02′46.57″ E). The leaf material was detached from stems, and the inflorescences were cut-off from stems before drying. A taxonomically identified voucher specimen (No Lazari D. 7347) has been deposited at the School of Pharmacy of the Aristotle University of Thessaloniki (Greece).

### 2.2. Extraction and Isolation

The air-dried flowers (328.17 g) and air-dried leaves (268.47 g) of *A. grandifollia* were successively extracted at room temperature with a mixture of solvents (petroleum ether: ether: MeOH/1:1:1) first and with methanol right after the extracts provided with the mixture of solvents were washed with brine. Then, the aqueous layer was re-extracted with ethyl acetate. The extraction procedure was the same as described previously [16]. The organic layer was dried over Na_2_SO_4_ and concentrated under reduced pressure to obtain a viscous mass (~8.23 g for the inflorescences and ~4.25 g for the leaves).

### 2.3. Compound Isolation

**Figure 1:**
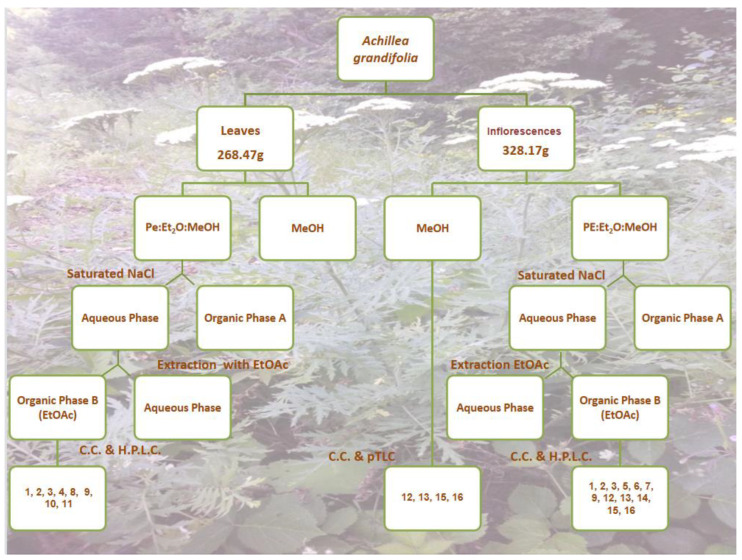
Schematic process of isolation of secondary metabolites from *Achillea grandifolia* with individual numbers referring to the isolated compounds. _(L)_: Leaves; _(I)_: Inflorescences.

The residue of the organic phase from the inflorescences (8.23 g) was subjected to VLC over silica gel (10 × 7 cm) being used as eluent mixtures of increasing polarity (Hexane-Ethyl acetate-Acetone-Methanol) to finally yield eleven fractions (A–L) (Figure 1, Table 1). The fraction D (770.3 mg) was submitted to CC on silica gel using CH_2_Cl_2_-MeOH mixtures of increasing polarity as eluents to provide thirteen fractions (DA–DN). The fraction DL (62.2 mg) was submitted to Sephadex LH-20 using MeOH (100%) as eluent to give three fractions (DLA-DLC). From these, fractions DLB (2.5 mg) was identified as compound **12** (Apigenin). The fraction E (509.1 mg) was submitted to CC on silica gel using CH_2_Cl_2_-MeOH mixtures of increasing polarity as eluents to give eleven fractions (EA–EL). A quantity (52.1 mg) of the fraction EG (176.4 mg) was further fractionated by semi-preparative HPLC (MeOH:H_2_O, 1:1, 1.50 mL/min), which allowed the isolation of compound **2** (rupicolin B) (t_R_ = 18.61 min, 10.9mg), compound **1** (rupicolin A) (t_R_ = 23.05 min, 5.8 mg), compound **13** (Luteolin) (t_R_ = 42.62 min, 0.9 mg), and compound **12** (apigenin) (t_R_ = 50.95 min, 2.1 mg). The fraction EK (24.7 mg) was submitted to C.C. on Sephadex LH-20 with MeOH as eluent to yield seven fractions (EKA–EKG). One of these fractions, EKF (7.2 mg) was identified as a specific compound **14** (eupatolitin). The fraction F (829.3 mg) was submitted to CC on silica gel using CH_2_Cl_2_-MeOH mixtures of increasing polarity as eluents to provide eleven fractions (FA–FL). The fraction FF (85.6 mg) was further fractionated by semipreparative HPLC (MeOH:H_2_O, 1:1, 1.50 mL/min), which allowed the isolation of compound **1** (rupicolin A) (t_R_ = 21.41 min, 6.8 mg). Also, 33.5 mg of the fraction FG (265.2 mg) were further fractionated by semi-preparative HPLC (MeOH:H_2_O, 1:1, 1.50 mL/min), which allowed the isolation of compound **2** (rupicolin B) (t_R_ = 19.04 min, 12.2 mg) and compound **1** (rupicolin A) (t_R_ = 23.25 min, 4.1 mg). The fraction FI (107.2 mg) was submitted to Sephadex LH-20 using MeOH (100%) as eluent to provide eight fractions (FIA–FIH). From these, the fraction FIH (4.8 mg) was identified as compound **13** (Luteolin). The fraction G (613.8 mg) was submitted to CC on silica gel using CH_2_Cl_2_-MeOH mixtures of increasing polarity as eluents to provide eight fractions (GA–GH). The fraction GD (65.0 mg) was further fractionated by semi-preparative HPLC (MeOH:H_2_O, 1:1, 1.50 mL/min), which allowed the isolation of compound **6** [(1S,2S,4αR,5R,8R,8αS)-decahydro-1,5,8-trihydroxy-4α,8-dimethyl–methylene-2-naphthaleneacetic acid methyl ester] (t_R_ = 18.12 min, 4.7 mg). A quantity (54.1 mg) of the fraction GG (144.8mg) was further fractionated by semi-preparative HPLC (MeOH:H_2_O, 1:1, 1.50 mL/min), which allowed the isolation of compound **3** (4S,6aS,9R,9aS,9bS)-4,6a,9-Trihydroxy-9-methyl-3,6-dimethylene-3a,4,5,6,6a,9, 9a,9b-octahydro-3H-azuleno[4,5-b]furan-2-one) (t_R_ = 12.28 min, 12.5 mg) and compound **7** (1β,3β,6α-Trihydroxycostic acid methyl ester) (t_R_ = 14.78 min, 3.5 mg). Fraction H (575.5 mg) was submitted to CC on silica gel using CH_2_Cl_2_-MeOH mixtures of increasing polarity as eluents to give nine fractions (HA–HI). From the fraction HF (125.1 mg), 42.5 mg were further fractionated by semi-preparative HPLC (MeOH:H_2_O, 1:1, 1.50 mL/min), which allowed the isolation of compound **5** (ridentin B) (t_R_ = 16.00 min, 4.8 mg).

The methanol extract was concentrated, and the residue (9.27 g) was dissolved in boiling water (H_2_O). The water-soluble fraction was filtered and extracted successively with diethyl-ether (Et_2_O), ethyl acetate (EtOAc), and n-butanol (n-BuOH). The ethyl acetate residue (1.35 g) was subjected to column chromatography on a Sephadex LH-20 using MeOH (100%) as eluent to provide several fractions (A–M). The fraction EF was submitted to CC on a Sephadex LH-20 with MeOH as eluent to yield twelve fractions (EFA–EFH) (Figure 1, Table 1). The fraction EFF (17.1 mg) was further purified on cellulose pTLC, which led to the isolation of compound **16** (luteolin7-*O*-glucopyranoside) (R_f_ = 0.28 on 30% acetic acid). The fraction EH (62.2 mg) was further purified on silica pTLC, which led to the isolation of compound **12** (apigenin) (R_f_ = 0.40 for CH_2_Cl_2_:MeOH:H_2_O / 90:10:1). The fraction EL (21.4 mg) was further purified on cellulose pTLC, which lead to the isolation of compound **13** (luteolin) (R_f_ = 0.18 for 30% acetic acid) and compound **15** (apigenin 7-*O*-glucopyranoside) (R_f_ = 0.67 for 30% acetic acid).

The residue of the organic phase from the leaves (4.25 g) was subjected to VLC over silica gel (10 × 7cm) using eluent mixtures of increasing polarity (Hexane-Ethyl acetate-Acetone-Methanol) to finally yield eleven fractions (A–L) (Figure 1, Table 1). The fraction D (241.7 mg) was submitted to CC on silica gel using CH_2_Cl_2_-MeOH mixtures of increasing polarity as eluents to provide sixteen fractions (DA–DQ). The fraction DI (58.0 mg) was further fractionated by semi-preparative HPLC (MeOH:H_2_O, 1:1, 1.20 mL/min), which allowed the isolation of compound **3** (4S,6aS,9R,9aS,9bS)-4,6a,9-Trihydroxy-9-methyl-3,6-dimethylene-3a,4,5,6,6a,9,9a,9b-octahydro-3H-azuleno[4,5-b]furan-2-one) (t_R_=11.85 min, 3.1 mg), compound **2** (rupicolin B) (t_R_ = 18.58 min, 10.8 mg), and compound **1** (rupicolin A) (t_R_ = 25.40 min, 5.4 mg). The fraction E (130.1 mg) was submitted to CC on silica gel using CH_2_Cl_2_-MeOH mixtures of increasing polarity as eluents to give twenty-five fractions (EA–EZ). The fraction EI (9.2 mg) was further fractionated by semi-preparative HPLC (MeOH:H2O, 1:1, 1.20 mL/min), which allowed the isolation of compound **11** (loliolide) (t_R_ = 20.46 min, 0.8 mg). The fraction EL (18.3 mg) was further fractionated by semi-preparative H.P.L.C. (MeOH:H_2_O, 1:1, 1.20mL/min), which allowed the isolation of compound **4** (artecalin) (t_R_ = 22.85 min, 2.2 mg). The fraction EO (24.4 mg) was further fractionated by semi-preparative HPLC (MeOH:H_2_O, 1:1, 1.20 mL/min), which allowed the isolation of compound **2** (rupicolin B) (t_R_ = 20.11min, 9.5mg) and compound **1** (rupicolin A) (t_R_ = 26.37 min, 4.3 mg). The fraction F (299.0 mg) was submitted to CC on silica gel using CH_2_Cl_2_-MeOH mixtures of increasing polarity as eluents to provide twenty-one fractions (FA–FV). The fraction FF (10.8 mg) was further fractionated by semi-preparative HPLC (MeOH:H_2_O, 1:1, 1.20 mL/min), which allowed the isolation of compound **10** (lingustolide A) (t_R_ = 13.71 min, 1.5 mg). The fraction FH (9.5 mg) was further fractionated by semi-preparative HPLC (MeOH:H_2_O, 1:1, 1.20 mL/min), which allowed the isolation of compound **8** (acrifolide) (t_R_ = 12.44 min, 1.3 mg). The fraction FL (44.3 mg) was further fractionated by semi-preparative HPLC (MeOH:H_2_O, 1:1, 1.20 mL/min), which allowed the isolation of compound **2** (rupicolin B) (t_R_ = 22.04 min, 6.0 mg). The fraction G was submitted to CC on silica gel using CH_2_Cl_2_-MeOH mixtures of increasing polarity as eluents to provide twenty-one fractions (GA–GV). From these, the fraction GH (8.3 mg) was identified as compound **9** (arteludovicinolide A). The fraction GQ (21.4 mg) was further fractionated by semi-preparative HPLC (MeOH:H_2_O, 1:1, 1.20 mL/min), which allowed the isolation of compound **3** (4S,6aS,9R,9aS,9bS)-4,6a,9-trihydroxy-9-methyl-3,6-dimethylene-3a,4,5,6,6a,9, 9a,9b-octahydro-3H-azuleno[4,5-b]furan-2-one) (t_R_ = 13.87 min, 4.3 mg).

### 2.4. Viability Assay

The glioma cell lines employed were the U87MG (glioma cell line, used as a reference in neuro-oncology) and T98G (glioblastoma multiforme cell line, also popular in neuro-oncology studies). The cell lines were exposed to rupicolin A and B at increased concentrations for the viability experiments (0–100 μΜ). Both compounds were dissolved in dimethyl sulfoxide (DMSO) before incubation. The final concentration of DMSO was below 0.1% in all cases (maximal concentration 0.05% DMSO). Viability calculation was performed as previously described [17]. Briefly, the 3-(4,5-dimethylthiazol-2-yl)-2,5-diphenyltetrazolium bromide (MTT, Sigma Life Sciences, Darmstadt, Germany) assay was used to evaluate cell viability. A total of 5000 cells were placed in 96-well plates, and, after 24 h, they were treated with rupicolin A and B at increasing concentrations for additional 48 h without medium change. After incubation, MTT was added. Following the manufacturer’s protocol, we performed colorimetric analysis of absorbance which is proportional to cell viability.

### 2.5. Cell Cycle Analysis

Cell cycle analysis was performed as described previously [18]. Cells (10^4^) were treated with rupicolin A and B at the IC50 value for 48 h. Equal amounts of cells were treated with plain culture media as a negative control. After being treated with trypsin, the cells were harvested, centrifuged, and then rinsed with phosphate-buffered saline solution (PBS) before being exposed to propidium iodide (PI) working solution (50 g/mL PI, 20 mg/mL RNase A, and 0.1% Triton X-100) for 20 min at 37 °C in the dark. Using a flow cytometer, information from the PI fluorescence was gathered to a total count of 10,000 nuclei (FACScalibur, BD Biosciences, San Jose, CA, USA). The cell cycle fractions G0/G1, S, G2/M, and G1/S were calculated using the CellQuest software from BD Biosciences.

## 3. Results

### 3.1. Isolated Compounds

**Figure 2:**
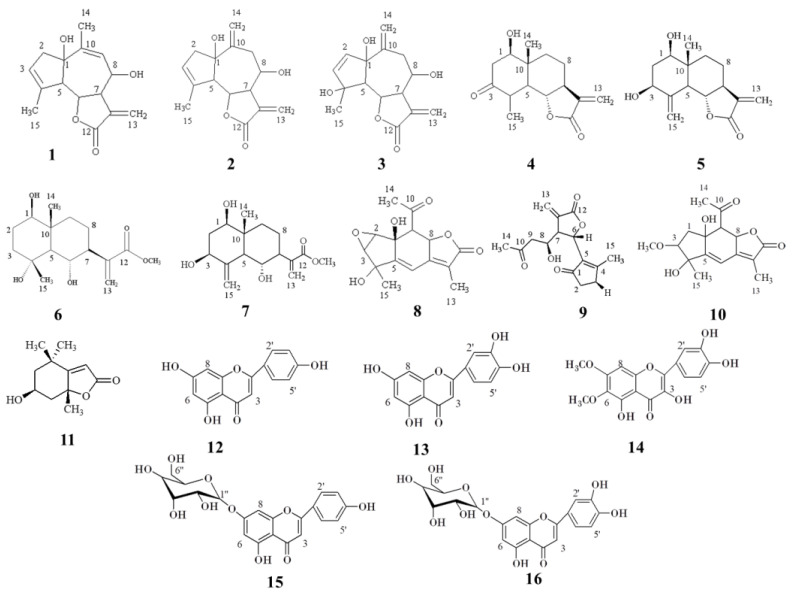
Isolated compounds from the leaves and inflorescences of Achillea grandifolia.

The column chromatography and semi-preparative HPLC of the methanol extract from the leaves and flowers of Achillea grandifolia led to the isolation of sixteen compounds. Eight compounds were isolated from the leaves (**1**, **2**, **3**, **4**, **8**, **9**, **10,** and **11**) and twelve from the inflorences (**1**, **2**, **3**, **5**, **6**, **7**, **9**, **12**, **13**, **14**, **15,** and **16**). The compounds were identified as rupicolin A (**1**), rupicolin B (**2**) [19], 4(4S,6aS,9R,9aS,9bS)-4,6a,9-Trihydroxy-9-methyl-3,6-dimethylene 3a,4,5,6,6a,9,9a, 9b-octahydro-3H-azuleno[4,5-b]furan-2-one (**3**) [20], artecalin (**4**) [21], ridentin B (**5**) [22], (1S,2S,4αR,5R,8R,8αS)-decahydro-1,5,8-trihydroxy-4α,8-dimethyl–methylene-2-naphthaleneacetic acid methyl ester (**6**) [23], 1β,3β,6α trihydroxycostic acid methyl ester (**7**) [24], acrifolide (**8**) [25], arteludovicinolide A (**9**) [26], lingustolide A (**10**) [21], loliolide (**11**) [27], apigenin (**12**) [28], luteolin (**13**) [29], eupatolitin (**14**) [30], apigenin-7-*O*-glucoside (**15**) [28], and luteolin 7-*O*-glucoside (**16**) [29]. The structures of the isolates are given in Figure 2 and were elucidated based on 1D and 2D NMR spectral analyses and by comparison of them with those found in the literature (see Tables S1–S16 and Figures S1–S52 in the supplementary materials).

### 3.2. Biological Activity

**Figure 3:**
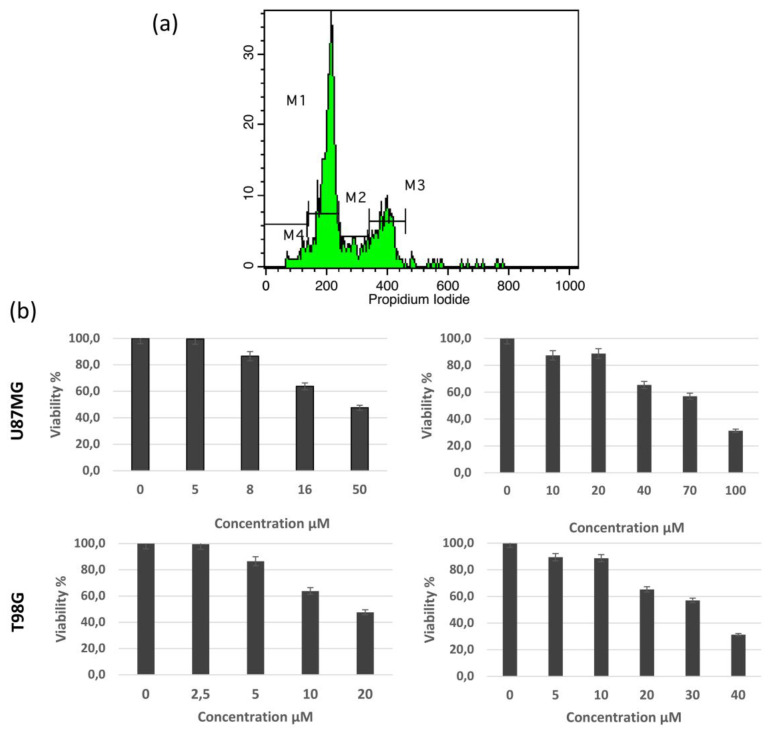
(**a**): Cell-cycle distribution assessed by flow cytometry in U87 and T98 glioblastoma cells. An indicative fluorescence distribution following PI staining is depicted (T98G cells treated with rupicolin B). The markers M1, M2, M3, and M4 correspond to the fraction of cells in G0, G1, S, and G2/M cell-cycle phases and subG1 cells, respectively. (**b**): Effect of different concentrations of rupicoline A (A) and rupicoline Β (B) on U87 and T98 cells. Different concentrations are presented in X-axis, while the difference in viability based on MTT colorimetric absorbance is presented in Y-axis.

We next analyzed the possible effects of the main compounds (**1**) and (**2**) on glioblastoma. The quantification of IC50 values were based on the quantification of MTT fluorescence, which is proportional to viable cell population. The concentration leading to a decrease in 50% of the standard MTT colorimetric absorbance for each cell line corresponds to the IC50. The concentrations used and the respective results are presented in Figure 3, panel B. The IC50 value of reduced viability during the 48-h treatment for rupicolin A **(1)** and rupicolin B **(2)** was 38 μM and 64 μM for the U87MG cells and 15 μM and 26 μM for the T98G cells, respectively. Both rupicolin A and B induced a G2/M cell cycle arrest. In the U87MG cells, the G2/M fraction was increased from 20.87% to 54.40% and 37.00% and in the T98G cells from 25.73% to 50.67% and 42.93% following rupicolin A and B treatment, respectively (Table 1). An increase in the S phase was also observed, which was more prominent in the case of the U87 line. Rupicolin A (**1**) and rupicolin B (**2**) in higher concentrations induced cytotoxic phenomena, thereby slightly inducing the subG1 cell population (Table 2 and Figure 3). A flow cytometric analysis of the DNA content with a guide for cell cycle fractions quantification is presented in Figure 3.

## 4. Discussion

The genus *Achillea* is well known for its richness in flavonoids. Many studies reveal that apigenin, luteolin, and their derivatives are the main flavonoids in polar extracts of the aerial parts of the plants, since they are detected in many species such as *Achillea millefolium* [31], *A. collina* [32], *A. sivasica* Çelik & Akpulat [33]. These results are in accordance with the current research on the isolation of compounds **12, 13, 14, 15,** and **16**.

All compounds referred herein were isolated from *A. grandifolia* for the first time. Moreover, according to our knowledge, this is the first report regarding the occurrence of compounds **3, 6, 11,** and **14** in any *Achillea* species. Further studies are needed to verify whether these compounds are species-specific or are to be found in other *Achillea* species as well.

Rupicolin A and B (**1** and **2**) have been isolated for the first time from the plant *Artemisia tripartita* Rydb. subsp. *rupicola* Beetle (Asteraceae) [19]. It has been suggested that the occurrence of rupicolins in many species of the genus *Achillea* can serve as a good chemotaxonomical marker [34]. In many cases, there is a co-occurrence of both rupicolin A and B (compounds **1** and **2**) in different *Achillea* species such as in *A. biebersteinii* Kotschy [35,36], *A. setacea* Waldst. & Kit. [37], *A. crithmifolia* Waldst. & Kit. [38], *A. clypeolata* Sm. [39], *A. chrysocoma* Friv., *A. coarctata* [40], and *A. clavennae* [34].

The name of compound **9** (arteludovicinolide-A) has been given after *Artemisia luboviciana* Nutt. (Asteraceae) was found to be the plant from which this component was isolated for the very first time [24]. Until now, this compound had also been isolated from *Achillea chrysocoma* [40], *A. coarctata* [41], and *A. millefolium* [26].

Acrifolide is a 1,2-seco-guaianoIide hemiacetal that has only been reported, to date, in *Achillea* species (compound **8**). The first report of this compound was in 2000 from the aerial parts of *A. crithmifollia* [42]. According to our knowledge, acrifolide has only been isolated from plants belonging to the genus *Achillea* such as *A. chrysocoma* [40] and *A. pseudopectinata* Janka [43].

Another seco-guaianolide derivative, namely, lingustolide-A (compound **10**), has been isolated from the leaves of the examined plant. This is a very rare compound, and its first report originates from *Achillea ligustica* All. (Asteraceae) [21]. Later, it has been isolated from *Achillea coarctata* [41] and *Artemisia argyi* H.Lév. & Vaniot [44].

Previous studies [45] have firstly described NMR data about the compound **7,** namely, 1β,3β,6α-Trihydroxycostic acid methyl ester, and the triacetate derivative of this natural product was isolated from *Artemisia rutifolia* Stephan ex Spreng. [46]. It should be noted, however, that this study provides the first report for this compound in a member of the genus *Achillea* i.e., *A. grandifolia*.

The compound **6,** namely, (1S,2S,4aR,5R,8R,8aS)-decahydro-1,5,8-trihydroxy-4a,8-dimethyl-methylene-2-naphthaleneacetic acid methyl ester, is also a very rare natural product, and this study is only the second to isolate this compound from plants, while the first report belongs to *Laurus nobilis* L. [23].

The first isolation of the eudesmanolide ridentin B (**5**) was performed from *Artemisia tripartita* subsp. *rupicola* [19], but it has also been reported from other plants of the same genus [47] such as *A. asiatica* Nakai ex Pump. [22], as well as from *Achillea coarctata* and *A. chrysocoma* [40].

Artecalin (**4**) is an eudesmanolide which has also been reported in other plants of the genus *Achillea*, such as *A. biebersteinii* [35], *A. coarctata* and *A. chrysocoma* [40], and *A. ligustica* [21]. Artecalin is a widely distributed sesquiterpene lactone in members of the Asteraceae family, since this compound has been isolated also from *Artemisia californica* Less., *A. tripartite* subsp. *rupicola* [48], and *Tanacetum santolina* C. Winkl [49], among others.

Loliolide (**11**) is a monoterpenoid hydroxylactone that is abundant in Asteraceae such as *Mantisalca salmantica* (L.) Briq. & Cavill. [50], *Artemisia integrifolia* L. [51], *Xanthium spinosum* L. [27], and *Gynura bicolor* (Roxb. ex Willd.) DC. [52], among others. Moreover, loliolide has been detected in the non-polar extracts (volatile oil and hexane extract) of *Achillea millefolium* [53] and *Achillea biebersteinii* [54].

Both rupicolin A and B are guaianolides. According to the literature, these types of sesquiterpene lactones exhibit various medicinal properties such as anthelmintic, antimicrobial, high anti-tumor, antifeedant, root-growth, and germination-inhibiting actions [55]. This report acted as a motive, and, for this reason, the isolated compounds **1** and **2** were also subjected to an anti-proliferative assay on two different glioblastoma cell lines, U87MG and T98G. It is known that natural substances have anti-glioma activity [18,56]. However, this is the first research that examined the anti-proliferative activity of rupicolin B. Notably, a G2/M cell cycle arrest coupled with an induction of the S phase fraction, especially in the case of U87MG cells, is known to be associated with the anti-glioma activity of natural compounds such as deglucohellebrin [18], thereby supporting a similar mechanism of action that may be associated with an anti-inflammatory effect and the action of NF-kB transcription factor. Previous studies have revealed that rupicolin A had a protective effect on iodixanol-induced cytotoxicity in LLC-PK1 cells at concentrations of 10 µM, with a cell survival rate of 75.1 ± 1.9% [15]. The current research offered evidence that natural substances exert anti-cancer effects, thereby suggesting that rupicolin A and B are promising candidates for further research, given the continuous need for novel therapies for the second cause of human mortality [57]. To our knowledge, there are only few reports related to the anti-tumour activities of sesquiterpene lactones [58,59,60], thus also suggesting that guaianolides such as rupicolin A and rupicolin B may exhibit potential antitumor activities against various tumor cell lines. Our research confirmed this potential activity by illustrating the anti-proliferative and cytotoxic action of these compounds in glioma cell lines. To date, there is only one study concerning the anti-tumor activity of rupicolin A in cultured renal tubular cells (LLC-PK1) [15], while there is no study for rupicolin B; the results herein confirmed the anti-tumor activity of rupicolin B for the first time. Furthermore, the results of the current study expand our knowledge on the action of both rupicolin A and rupicolin B on the cell cycle distribution of cancer cells.

## 5. Conclusions

The phytochemical investigation of *Achillea grandifolia* proved that this species is a rich source of sesquiterpene lactones and flavonoids. We extracted and characterized sixteen known compounds from the inflorescences and leaves of *A grandifolia*. This is the first study leading to the isolation of non-volatile terpenoid compounds from the aerial part of this species of the genus *Achillea*. The most abundant compounds were rupicolin A (**1**) and B (**2**). These compounds were isolated from both the inflorescences and leaves of the species under study. Furthermore, these two main compounds of *A. grandifolia* were also examined for their anti-proliferative effect on U87MG and T98G glioblastoma cell lines. The preliminary results of the treatment of glioblastoma cell lines with A and B indicate that they have the potential to support novel anti-cancer therapies. Our current studies focus on the validation of the findings reported herein and on the investigation of the mechanism of their biological action with the aim to facilitate their establishment as possible anti-glioma agents.

## Figures and Tables

**Table 1:** Different compounds isolated from fractions of leaves (_L_) and inflorescences (_I_) of *Achillea grandifolia*.

Isolated in Fractions	Compound Number	Compound Name
DI_(L)_, EG_(F)_, EO_(I)_, FF_(I)_, FG_(I)_	**1**	Rupicolin A
DI_(L)_, EG_(I)_, EO_(L)_, FL_(L)_, FG_(I)_	**2**	Rupicolin B
DI_(L)_, GG_(I)_, GQ_(L)_	**3**	4S,6aS,9R,9aS,9bS)-4,6a,9-Trihydroxy-9-methyl-3,6-dimethylene-3a,4,5,6,6a,9,9a,9b-octahydro-3H-azuleno[4,5-b]furan-2-one)
EL_(L)_	**4**	Artecalin
FH_(I)_	**5**	Ridentin B
GD_(I)_	**6**	(1S,2S,4αR,5R,8R,8αS)-decahydro-1,5,8-trihydroxy-4α,8-dimethyl–methylene-2-naphthaleneacetic acid methyl ester
GG_(I)_	**7**	1β,3β,6α-Trihydroxycostic acid methyl ester
FH_(L)_	**8**	Acrifolide
GH_(L)_	**9**	Arteludovicinolide A
FF_(L)_	**10**	Lingustolide A
EI_(L)_	**11**	Loliolide
DLB_(I)_, EG_(I)_, EH_(L)_	**12**	Apigenin
EG_(I)_, EL_(L)_, FIH_(I)_	**13**	Luteolin
EFK_(F)_	**14**	Eupatolitin
EL_(L)_	**15**	Apigenin 7-O-glucopyranoside
EFF_(L)_	**16**	Luteolin 7-O-glucopyranoside

**Table 2:** Cell-cycle distribution assessed by flow cytometry in U87 and T98 glioblastoma cells. In each case, IC50 values were used for incubation.

Cell Line	U87MG			T98G		
Cell Cycle Phases	Control	Rupicolin A	Rupicolin B	Control	Rupicolin A	Rupicolin B
G_1_	64.73	15.87	23.60	58.27	19.27	32.60
S	9.20	24.27	37.60	13.67	24.13	17.20
G_2_/M	20.87	54.40	37.00	25.73	50.67	42.93
subG_1_	3.13	3.20	3.40	0.80	2.40	4.07

## Data Availability

All data referred to or generated in this study are included in tables or figures and are available upon request.

## Supplementary Materials

The following supporting information can be downloaded at: https://www.mdpi.com/article/10.3390/pharmaceutics15051383/s1, Supplementary material Note S1: Eluents used in the vacuum liquid chromatography (VLC) experiment; Figure S1: ^1^H-NMR spectrum of compound **1** (CDCl_3_, 500MHz); Figure S2: ^1^H-NMR full spectrum (A) and parts thereof (B) compound **1** (CD_3_OD, 500MHz); Figure S3: gDQCOSY spectrum of compound **1** (CD_3_OD, 500 MHz); Figure S4: ^13^C-NMR spectrum of compound **1** (CD_3_OD, 125MHz); Figure S5: gHSQCAD spectrum of compound **1** (CD_3_OD, 500 MHz); Figure S6: ^1^H-NMR full spectrum (A) and parts thereof (B) of compound **2** (CD_3_OD, 500MHz); Figure S7: gDQCOSY spectrum of compound **2** (CD_3_OD, 500 MHz); Figure S8: ^1^H-NMR full spectrum (A) and parts thereof (B) of compound **3** (CD_3_OD, 500MHz); Figure S9: gDQCOSY spectrum of compound **3** (CD_3_OD, 500 MHz); Figure S10: ^13^C-NMR spectrum of compound **3** (CD_3_OD, 125MHz); Figure S11: gHSQCAD spectrum of compound **3** (CD_3_OD, 500 MHz); Figure S12: gHMBCAD spectrum of compound **3** (CD_3_OD, 500 MHz); Figure S13: ^1^H-NMR full spectrum (A) and parts thereof (B) of compound **4** (CD_3_OD, 500MHz); Figure S14: ^13^C-NMR spectrum of compound **4** (CD_3_OD, 125MHz); Figure S15: gDQCOSY spectrum of compound **4** (CD_3_OD, 500 MHz); Figure S16: gHSQCAD spectrum of compound **4** (CD_3_OD, 500 MHz); Figure S17: gHMBCAD spectrum of compound **4** (CD_3_OD, 500 MHz); Figure S18: ^1^H-NMR full spectrum (A) and parts thereof (B) of compound **5** (CD_3_OD, 500MHz); Figure S19: gDQCOSY spectrum of compound **5** (CD_3_OD, 500 MHz); Figure S20: ^13^C-NMR spectrum of compound **5** (CD_3_OD, 125MHz); Figure S21: ^1^H-NMR full spectrum (A) and parts thereof (B) of compound **6** (CD_3_OD, 500MHz); Figure S22: gDQCOSY spectrum of compound **6** (CD_3_OD, 500 MHz); Figure S23: ^13^C-NMR spectrum of compound **6** (CD_3_OD, 125MHz); Figure S24: gHSQCAD spectrum of compound **6** (CD_3_OD, 500 MHz); Figure S25: gHMBCAD spectrum of compound **6** (CD_3_OD, 500 MHz); Figure S26: ^1^H-NMR full spectrum (A) and parts thereof (B) of compound **7** (CD_3_OD, 500MHz); Figure S27: gDQCOSY spectrum of compound 7 (CD_3_OD, 500 MHz); Figure S28: ^13^C-NMR spectrum of compound **7** (CD_3_OD, 125MHz); Figure S29: gHSQCAD spectrum of compound **7** (CD_3_OD, 500 MHz); Figure S30: gHMBCAD spectrum of compound **7** (CD_3_OD, 500 MHz); Figure S31: ^1^H-NMR spectrum of compound **8** (CD_3_OD, 500MHz); Figure S32: gDQCOSY spectrum of compound **8** (CD_3_OD, 500 MHz); Figure S33: ^13^C-NMR spectrum of compound **8** (CD_3_OD, 125MHz); Figure S34: gHSQCAD spectrum of compound **8** (CD_3_OD, 500 MHz); Figure S35: gHMBCAD spectrum of compound **8** (CD_3_OD, 500 MHz); Figure S36: ^1^H-NMR full spectrum (A) and parts thereof (B) of compound **9** (CD_3_OD, 500MHz); Figure S37: gDQCOSY spectrum of compound **9** (CD_3_OD, 500 MHz); Figure S38: ^13^C-NMR spectrum of compound **9** (CD_3_OD, 125MHz); Figure S39: gHSQCAD spectrum of compound **9** (CD_3_OD, 500 MHz); Figure S40: ^1^H-NMR spectrum of compound **10** (CD_3_OD, 500MHz); Figure S41: gDQCOSY spectrum of compound **10** (CD_3_OD, 500 MHz); Figure S42: ^1^H-NMR spectrum of compound **11** (CD_3_OD, 500MHz); Figure S43: gDQCOSY spectrum of compound **11** (CD_3_OD, 500 MHz); Figure S44: ^13^C-NMR spectrum of compound **11** (CD_3_OD, 125MHz); Figure S45: gHSQCAD spectrum of compound **11** (CD_3_OD, 500 MHz); Figure S46: gHMBCAD spectrum of compound **11** (CD_3_OD, 500 MHz); Figure S47: ^1^H-NMR spectrum of compound **12** (CD_3_OD, 500MHz); Figure S48: ^1^H-NMR spectrum of compound **13** (CD_3_OD, 500MHz); Figure S49: ^1^H-NMR spectrum of compound **14** (CD_3_OD, 500MHz); Figure S50: ^1^H-NMR spectrum of compound **15** (CD_3_OD, 500MHz); Figure S51: gDQCOSY spectrum of compound **15** (CD_3_OD, 500 MHz); Figure S52: ^1^H-NMR spectrum of compound **16** (CD_3_OD, 500MHz); Table S1: ^1^H and ^13^C NMR of compound **1** (CD_3_OD, 500 MHz); **Table S2**: ^1^H and ^13^C NMR of compound **2** (CD_3_OD, 500 MHz); Table S3: ^1^H and ^13^C NMR of compound **3** (CD_3_OD, 500 MHz); **Table S4**: ^1^H and ^13^C NMR of compound **4** (CD_3_OD, 500 MHz); Table S5: ^1^H and ^13^C NMR of compound **5** (CD_3_OD, 500 MHz); Table S6: ^1^H and ^13^C NMR of compound **6** (CD_3_OD, 500 MHz); **Table S7**: ^1^H and ^13^C NMR of compound **7** (CD_3_OD, 500 MHz); Table S8: ^1^H and ^13^C NMR of compound **8** (CD_3_OD, 500 MHz); Table S9: ^1^H and ^13^C NMR of compound **9** (CD_3_OD, 500 MHz); Table S10: ^1^H and ^13^C NMR of compound **10** (CD_3_OD, 500 MHz); Table S11: ^1^H and ^13^C NMR of compound **11** (CD_3_OD, 500 MHz); **Table S12**: ^1^H NMR of compound **12** (CD_3_OD, 500 MHz); Table S13: ^1^H NMR of compound **13** (CD_3_OD, 500 MHz); Table S14: ^1^H NMR of compound **14** (CD_3_OD, 500 MHz); Table S15: ^1^H NMR of compound **15** (CD_3_OD, 500 MHz); Table S16: ^1^H NMR of compound **16** (CD_3_OD, 500 MHz).
