# Supplementary material for: Isolation of Secondary Metabolites from Achillea grandifolia Friv. (Asteraceae) and Main Compounds’ Effects on a Glioblastoma Cellular Model
Source: Pharmaceutics. 2023 Apr 30;15(5):1383. doi: 10.3390/pharmaceutics15051383 (PMC10224520; doi:10.3390/pharmaceutics15051383)
Supplement: Supplementary file 1 [file pharmaceutics-15-01383-s001.zip › pharmaceutics-2282741-supplementary.pdf]

pharmaceutics

MDPI

Supplementary Materials

Isolation of secondary metabolites from *Achillea grandifolia* Friv. (Asteraceae) and main compounds' effects on a glioblastoma cellular model

Olga S. Tsiftsoglou [1], Nikos Krigas [2,*], Christos Gounaris [1], Christina Papitsa [1], Maria Nanouli [1], Evrysthenis Vartholomatos [3], George Markopoulos [3,4], Rafaela Isyhou [3], George Alexiou [3,5] and Diamanto Lazari [1,*]

Note S1: Eluents used in the vacuum liquid chromatography (VLC) experiment.

The solvents used in the experiments was diethyl ether (J.T. Baker 8254), aceton (Merck 1.00014.2511, CarloErba 400974), Dichloromethane (Panreac 131254, Merck 1.06050.2500, J.T. Baker 7053), hexane (J.T. Baker 8044, Riedel-de Haen 32293), sulfuric acid 96% (Panreac 131058), methanol(Merck 1.06009.2511, Merck 1.06007.2500, Panreac 131091), Ethyl acetate (Panreac 131318), acetic acid (J.T. Baker 6152) and pertoleum ether (Carlo Erba 447832, Panreac 131315).

The organic phase from the leaves of *Achillea grandifolia* (4.25 g) was subjected to VLC over silica gel (10 × 7cm) using as eluent mixtures of increasing polarity (Hexane-Ethyl acetate-Acetone-Methanol) to yield finally eleven fractions (A-L)

GRAN-A (32,2mg), PE 100%
GRAN-B (4,2mg) PE: EtOAc 75:25
GRAN-C (176,3mg) PE: EtOAc 50:50
GRAN-D (241,7mg) PE: EtOAc 25:75
GRAN-E (130,1mg) EtOAc 100%
GRAN-F (299,0mg) EtOAc: Ac 90:10
GRAN-G (170,9mg) EtOAc: Ac 75:25
GRAN-H (253,4mg) Ac 100%
GRAN-I (792,2mg) MeOH 100%
GRAN-K (2,0737g) MeOH 100%
GRAN-L (65,8mg) MeOH 100%

The organic phase from the inflorescences of *Achillea grandifolia* (8.23 g) was subjected to VLC over silica gel (10x7cm) using as eluent mixtures of increasing polarity (Hexane-Ethyl acetate-Acetone-Methanol) to yield finally eleven fractions (A-L)

GAF-A (128.3mg), He 100%
GAF-B (11.1mg), He:EtOAc 75:25
GAF-C (523.8mg), He:EtOAc 50:50
GAF-D (770.3mg), He:EtOAc 25:75
GAF-E (509.1mg), EtOAc 100%
GAF-F (829.3mg), EtOAc:Ac 90:10
GAF-G (613.8mg), EtOAc:Ac 75:25
GAF-H (575.5mg), Ac 100%
GAF-I (416.7mg), M 100%
GAF-K (1.8g), M 100%
GAF-L (68.4mg), M 100%

Table S1. ^{1}H and ^{13}C NMR of compound **1** (CD_3OD, 500 MHz).

No	δ_C	Type C	δ_H	H	J (Hz)
1	82.4	HO-C	-	-	-
2	45.8	CH	2.56	2	br s

3	122.7	=CH	5.47	1	s
4	141.3	C	-	-	-
5	64.7	CH	2.69	-	d (J = 11.0)
6	79.3	CH	3.95	1	dd (J = 11.0, 9.0)
7	49.7	CH	3.09	1	m
8	70.2	HO-CH	4.10	1	br d (J = 10.5)
9	127.1	CH_2	5.44	1	s
10	138.3	C	-	-	-
11	137.3	C	-	-	-
12	170.7	C=O	-	-	-
13a	123.1	CH_2	6.21	1	o.s
13b			6.21	1	o.s
14	23.2	CH_3	1.90	3	s
15	16.7	CH_3	1.90	3	s

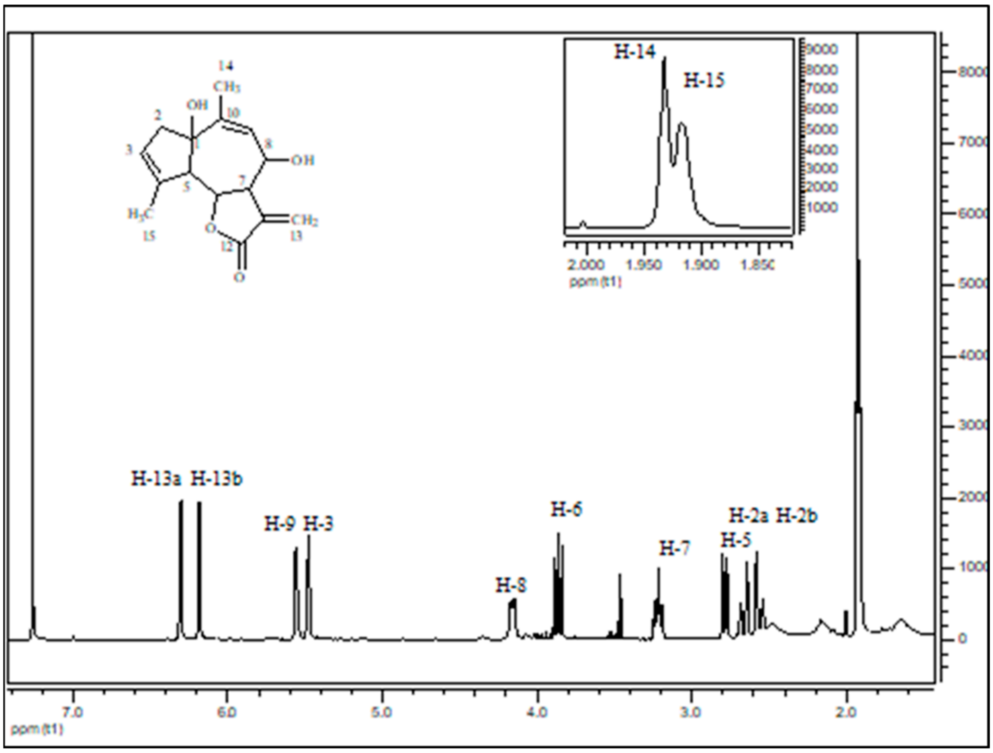

Figure S1. ^{1}H-NMR spectrum of compound **1** ($CDCl_3$, 500MHz).

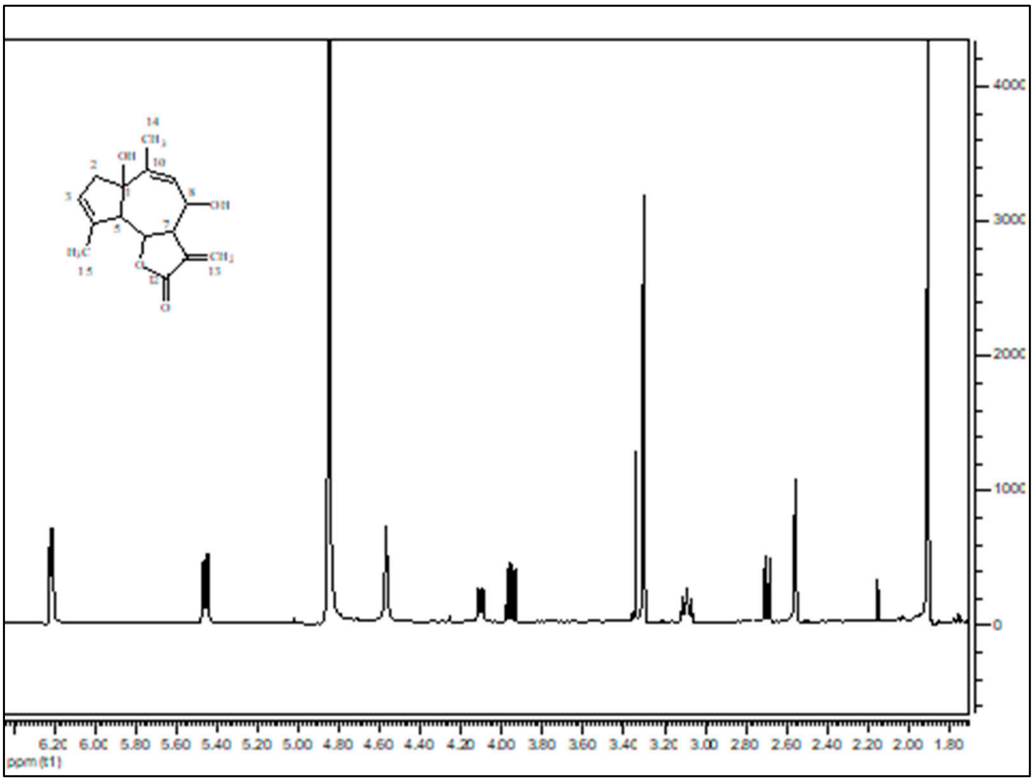

(A)

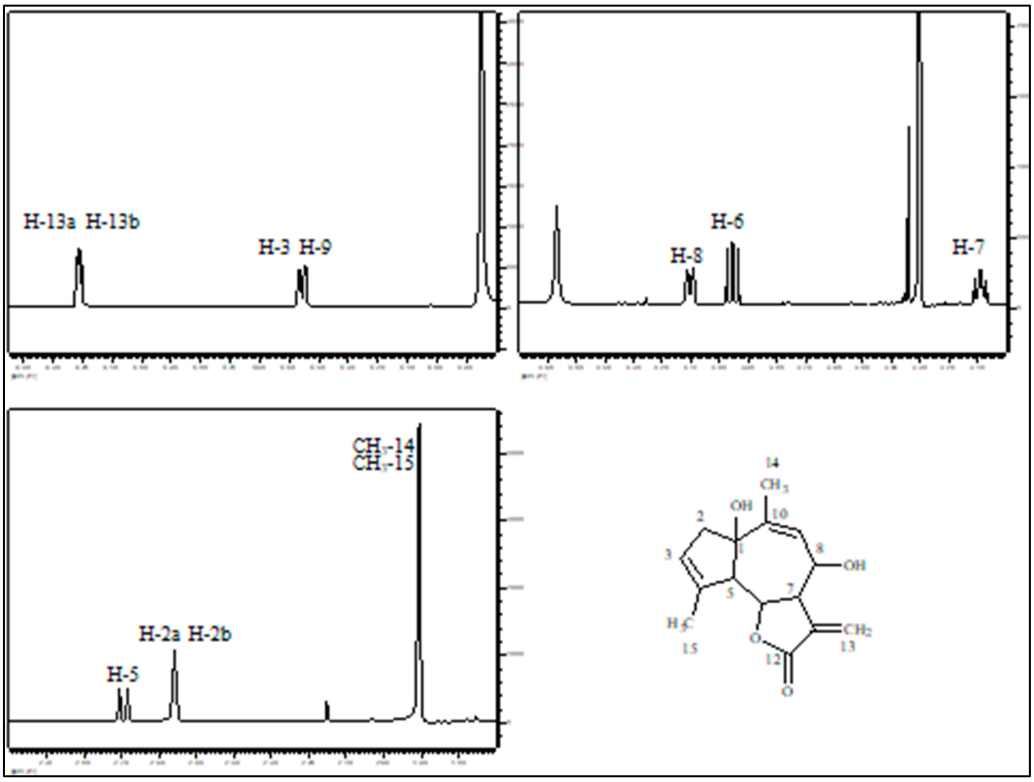

(B)

Figure S2. ^{1}H-NMR full spectrum (A) and parts thereof (B) of compound **1** (CD_3OD, 500MHz)

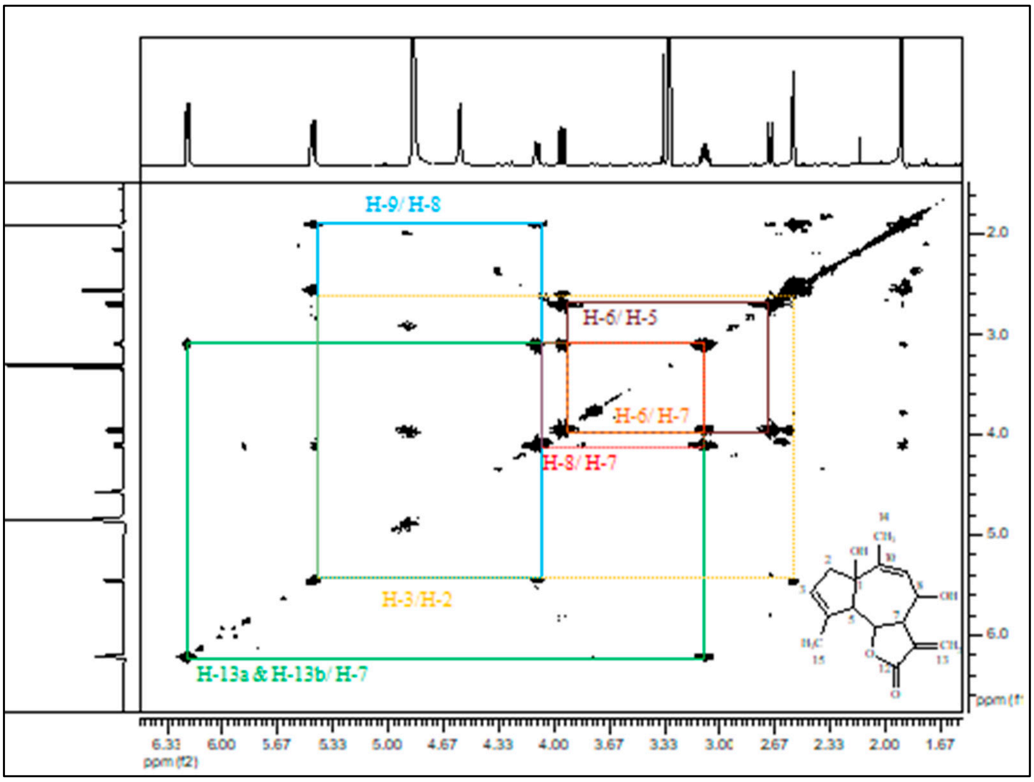

Figure S3. gDQCOSY spectrum of compound **1** (CD3OD, 500MHz).

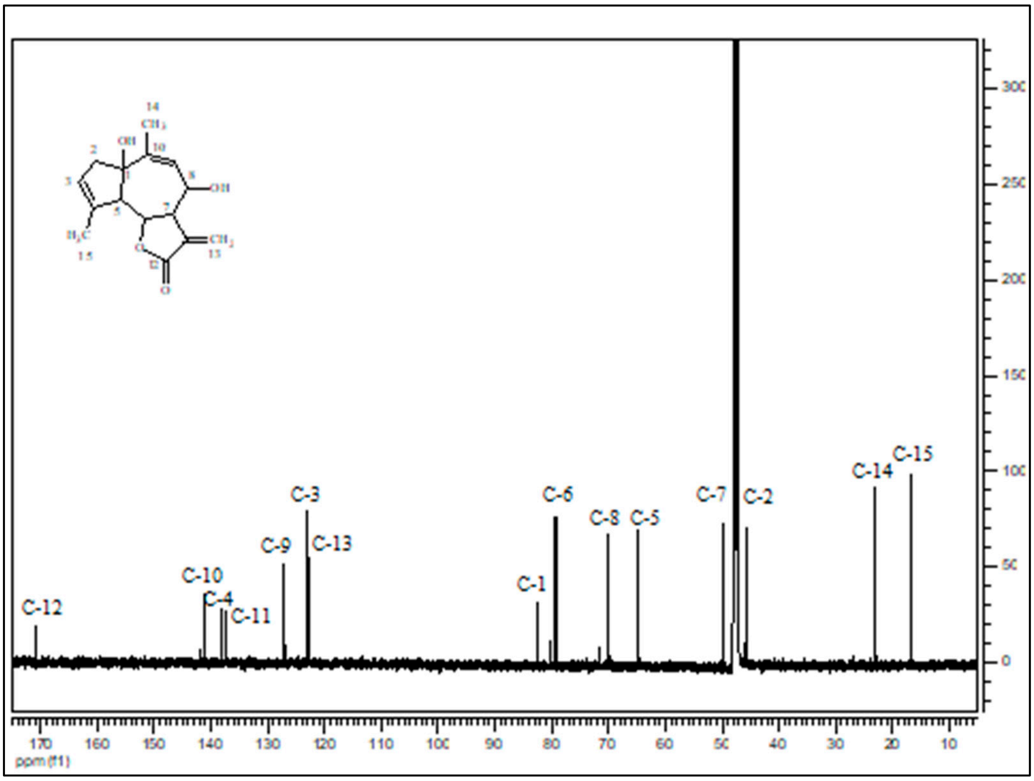

Figure S4. ^{13}C-NMR spectrum of compound **1** (CD3OD, 125MHz).

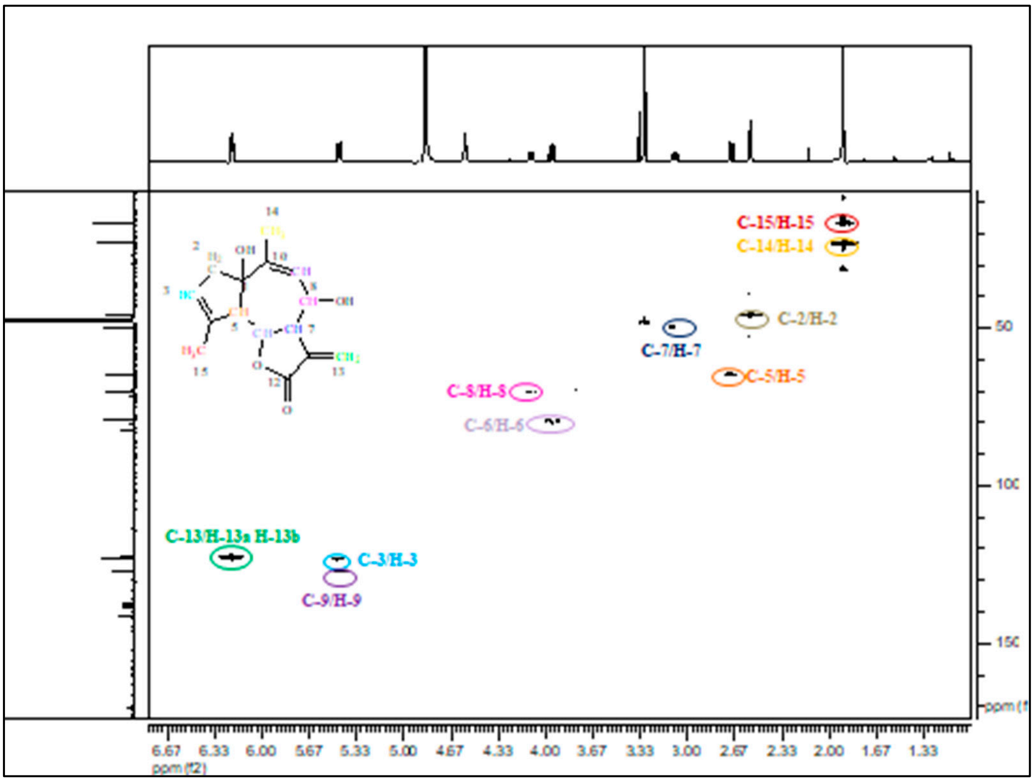

Figure S5. gHSQCAD spectrum of compound **1** (CD_3OD, 500MHz).

Table S2. ^{1}H and ^{13}C NMR of compound **2** (CD_3OD, 500 MHz).

No	δC	Type C	δH	H	J (Hz)
1	84.1	HO-CH	-	-	-
2a	44.8	CH	2.84	1	br d (J = 17.0)
2b			2.28	1	d (J = 16.5)
3	124.5	=CH	5.51	1	s
4	139.9	C	-	-	-
5	64.5	CH	2.61	-	d (J = 9.5)
6	80.3	CH	3.91	1	dd (J = 10.5, 9.5)
7	50.6	CH	3.00	1	m
8	72.3	HO-CH	3.79	1	m
9a	40.1	CH_2	2.61	1	dd (J = 13.6, 6.5)
9b			2.49	1	dd (J = 13.6, 5.0)
10	145.9	C	-	-	-
11	138.5	C	-	-	-
12	170.9	C=O	-	-	-
13a	121.9	CH_2	6.15	1	o.s
13b			6.15	1	o.s
14a	115.2	=CH_2	5.30	1	s
14b			5.07	1	s
15	16.4	CH_3	1.87	3	s

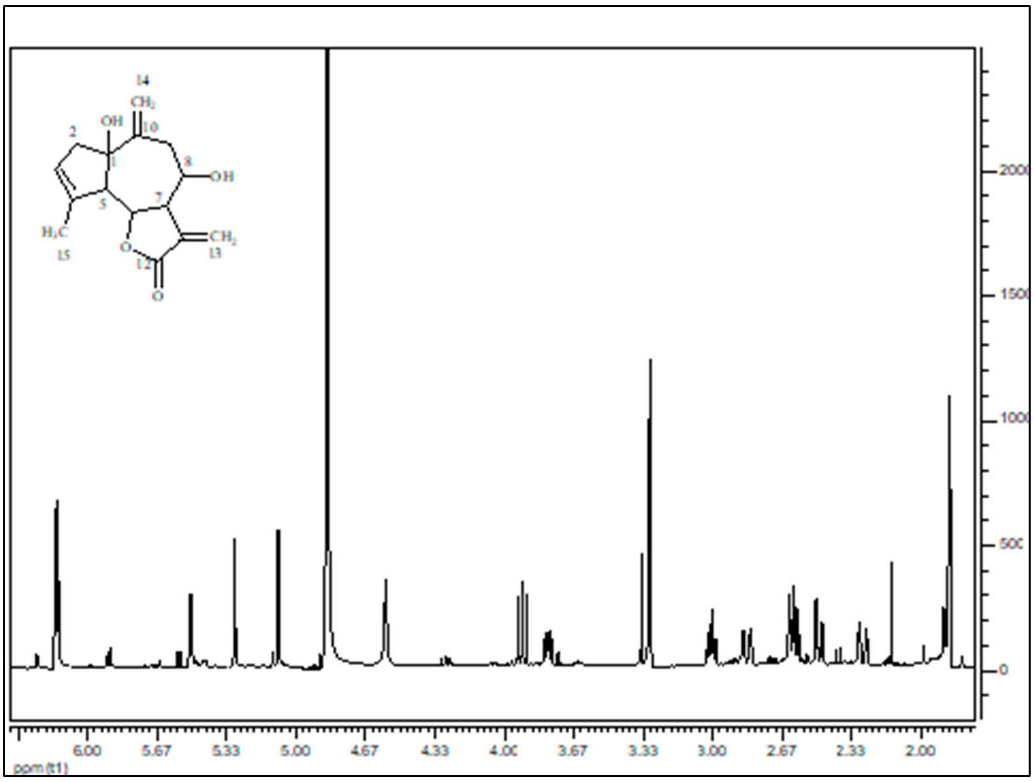

(A)

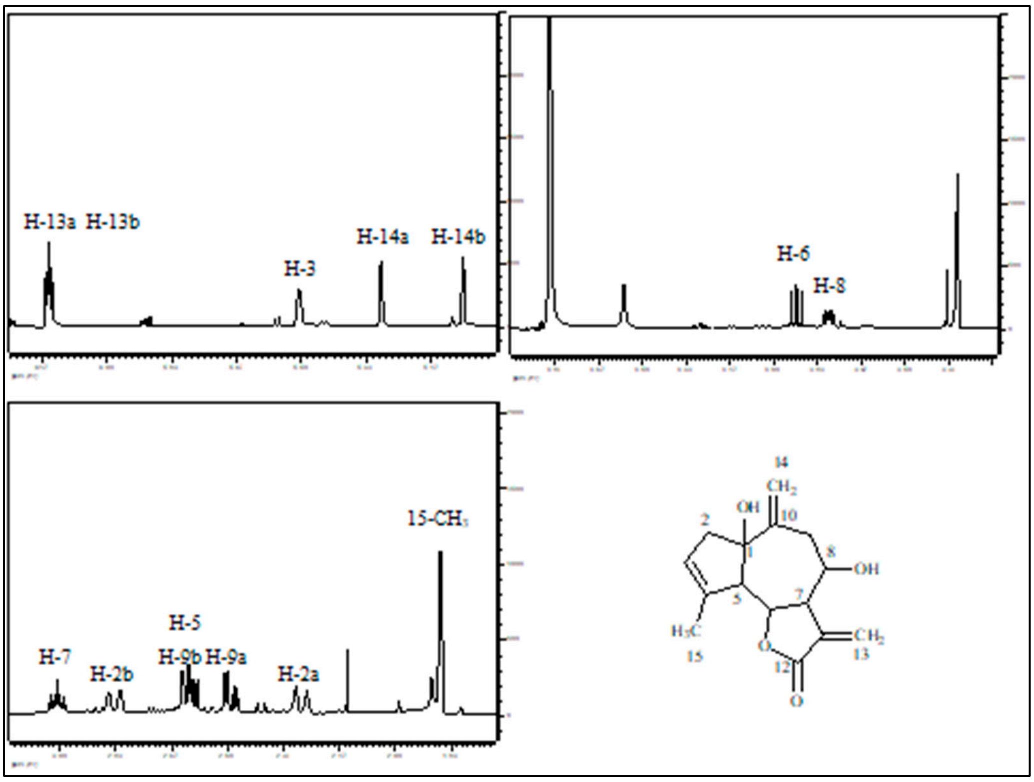

(B)

Figure S6. ^{1}H-NMR full spectrum (A) and parts thereof (B) of compound **2** (CD$_3$OD, 500MHz).

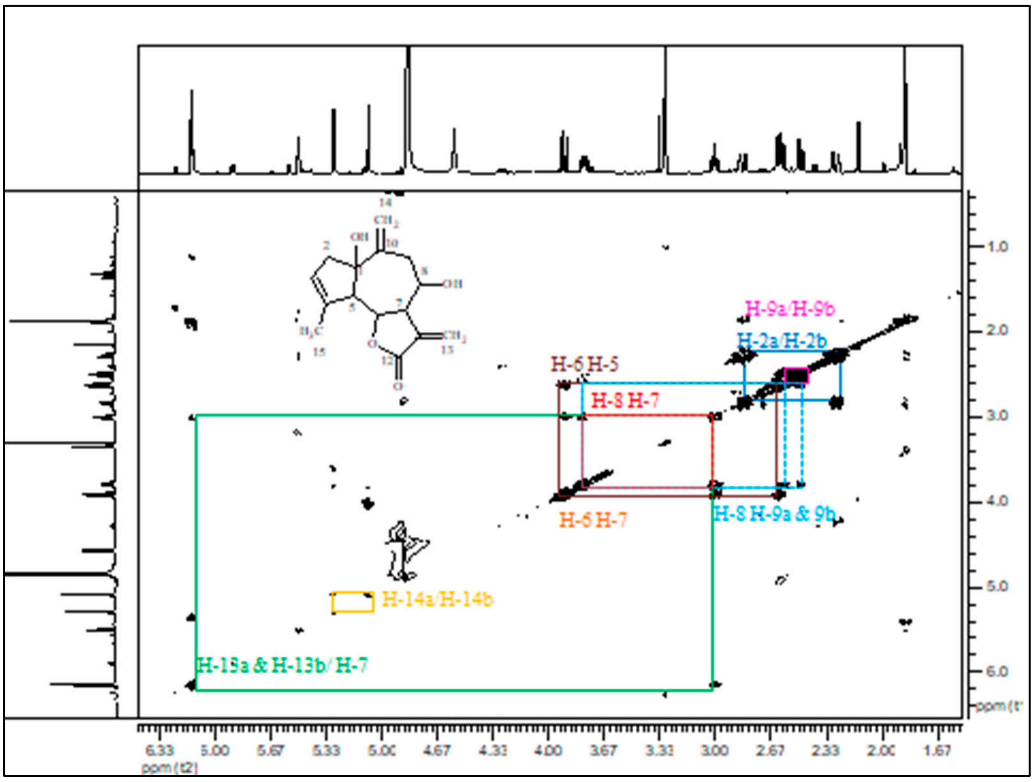

Figure S7. gDQCOSY spectrum of compound **2** (CD$_3$OD, 500 MHz).

Table S3. ^{1}H and ^{13}C NMR of compound **3** (CD_3OD, 500 MHz).

No	δC	Type C	δH	H	J (Hz)
1	81.7	HO-CH	-	-	-
2	137.2	=CH	5.87	1	d (J = 5.5)
3	139.9	=CH	5.56	1	d (J = 5.5)
4	145.6	HO-C	-	-	-
5	64.9	CH	2.58	1	d (J = 9.5)
6	78.7	O-CH	4.15	1	dd (J = 11.5, 9.0)
7	50.6	CH	3.37	1	m
8	73.0	HO-CH	3.70	1	m
9a	40.9	CH_2	2.55	1	dd (J = 12.0, 5.5)
9b			2.97	1	dd (J = 12.0, 1.5)
10	84.8	C	-	-	-
11	139.8	C	-	-	-
12	171.2	C=O	-	-	-
13a	134.3	$=CH_2$	6.32	1	dd (J = 3.0, 1.5)
13b			6.24	1	dd (J = 3.0, 1.5)
14a	124.6	$=CH_2$	5.04	1	s
14b				1	o.s
15	22.6	CH_3	1.29	3	s

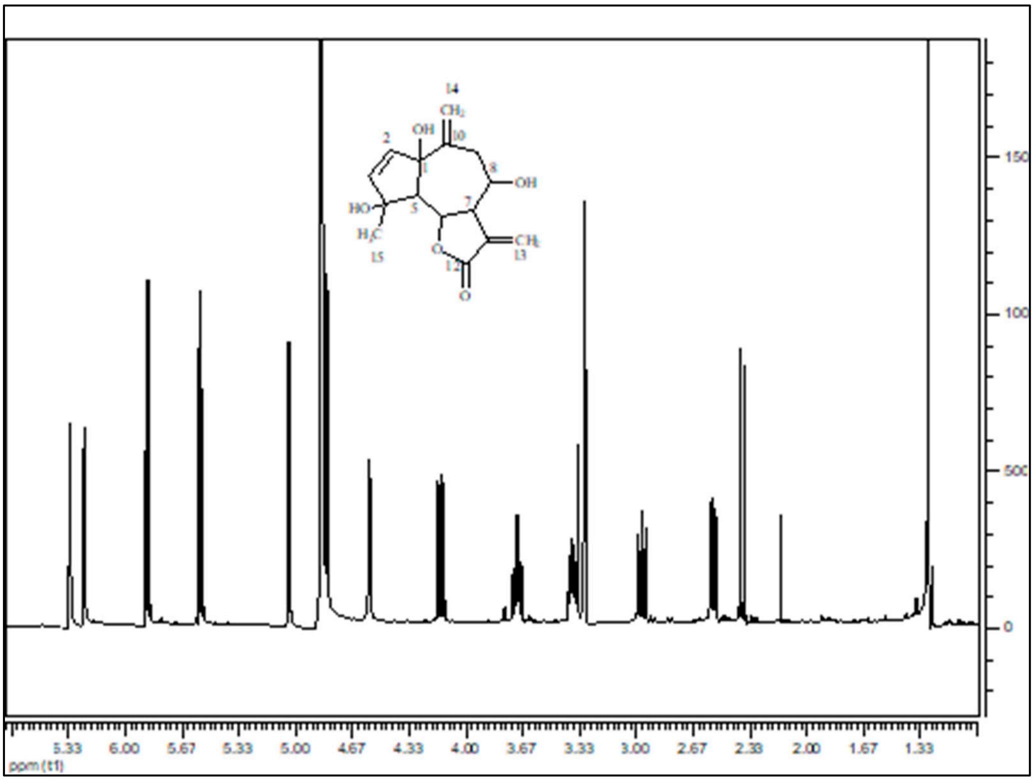

(A)

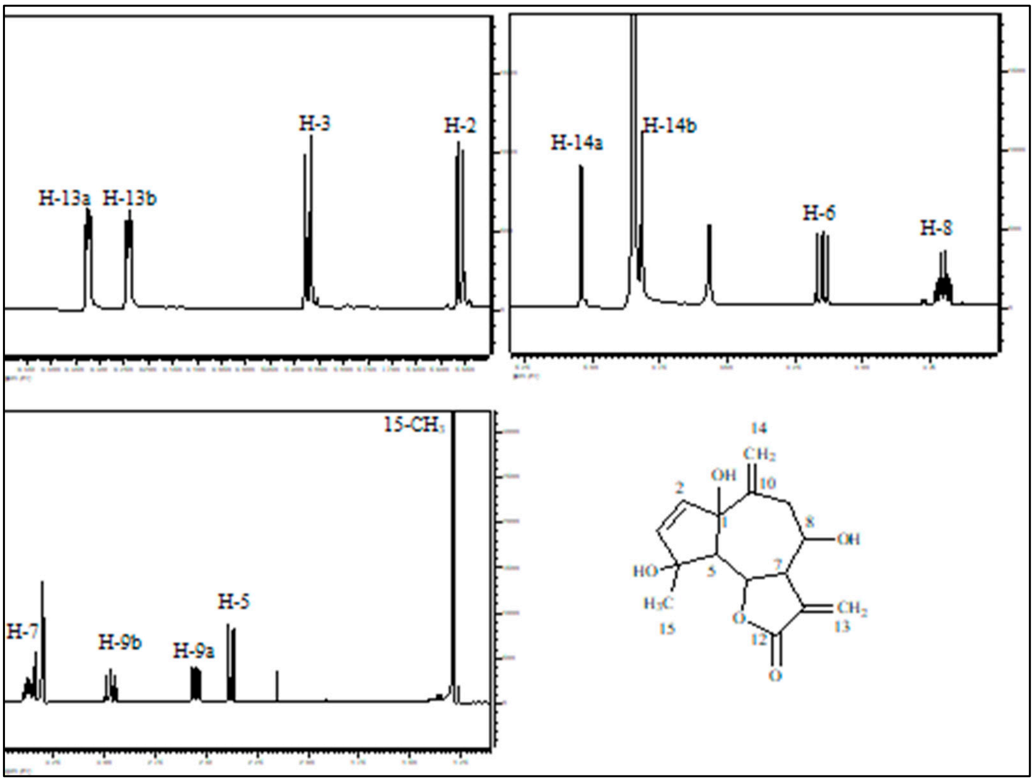

(B)

Figure S8. ^{1}H-NMR full spectrum (A) and parts thereof (B) of compound **3** (CD$_3$OD, 500MHz).

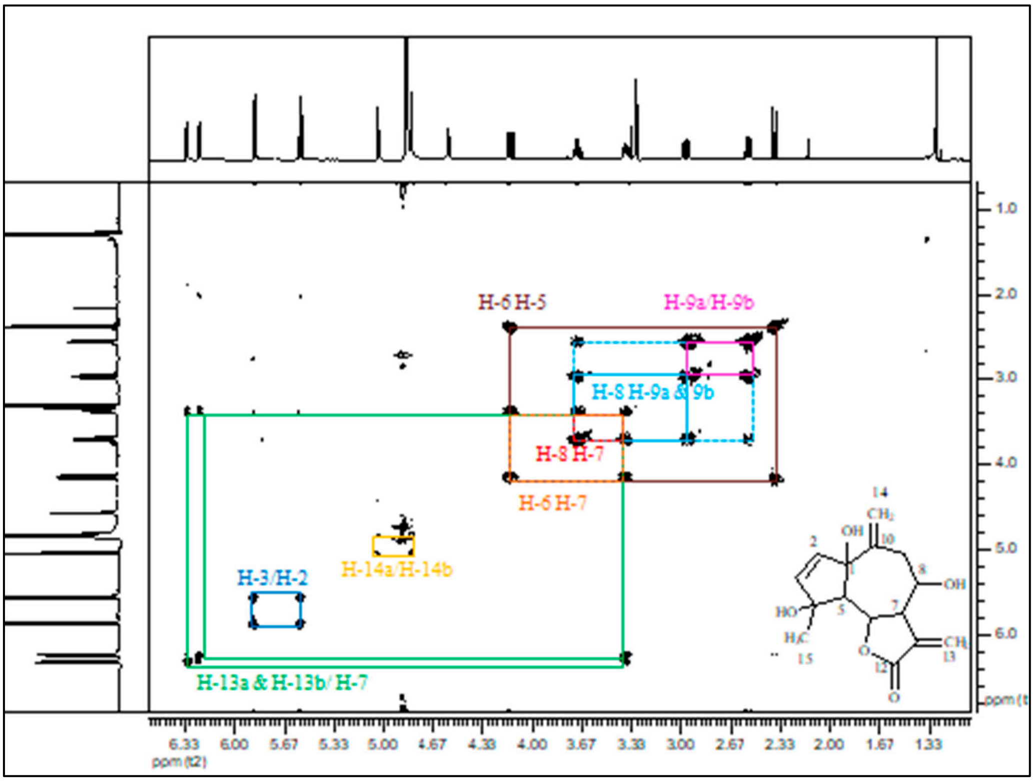

Figure S9. gDQCOSY spectrum of compound **3** (CD$_3$OD, 500 MHz).

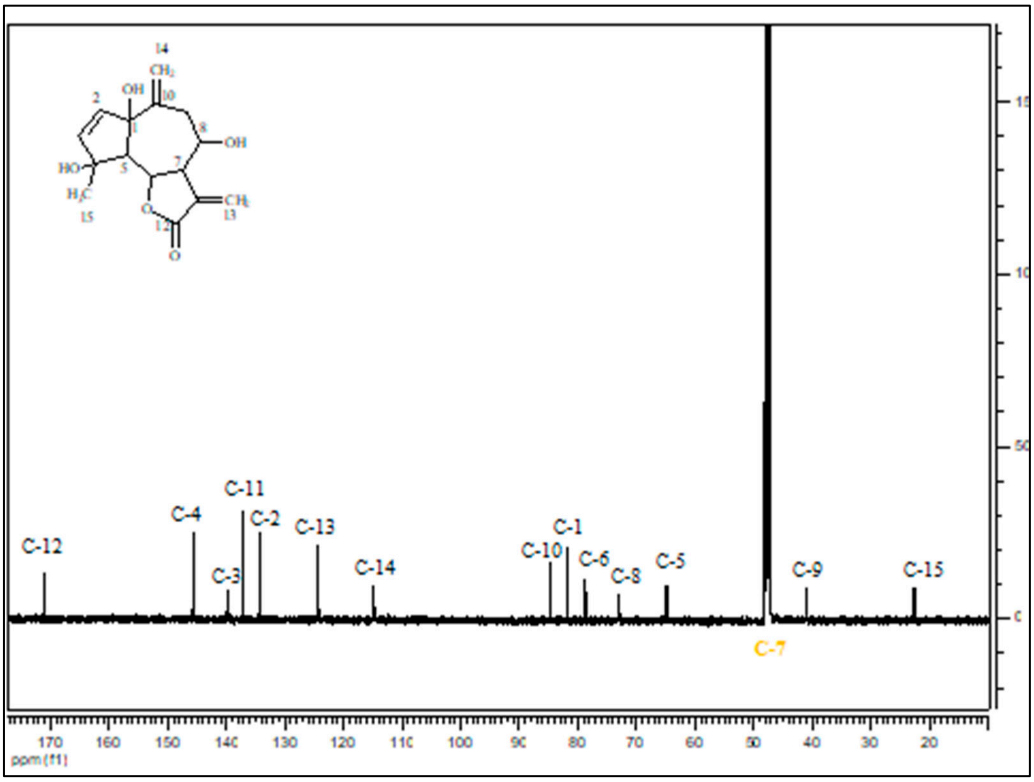

Figure S10. ^{13}C-NMR spectrum of compound **3** (CD_3OD, 125MHz).

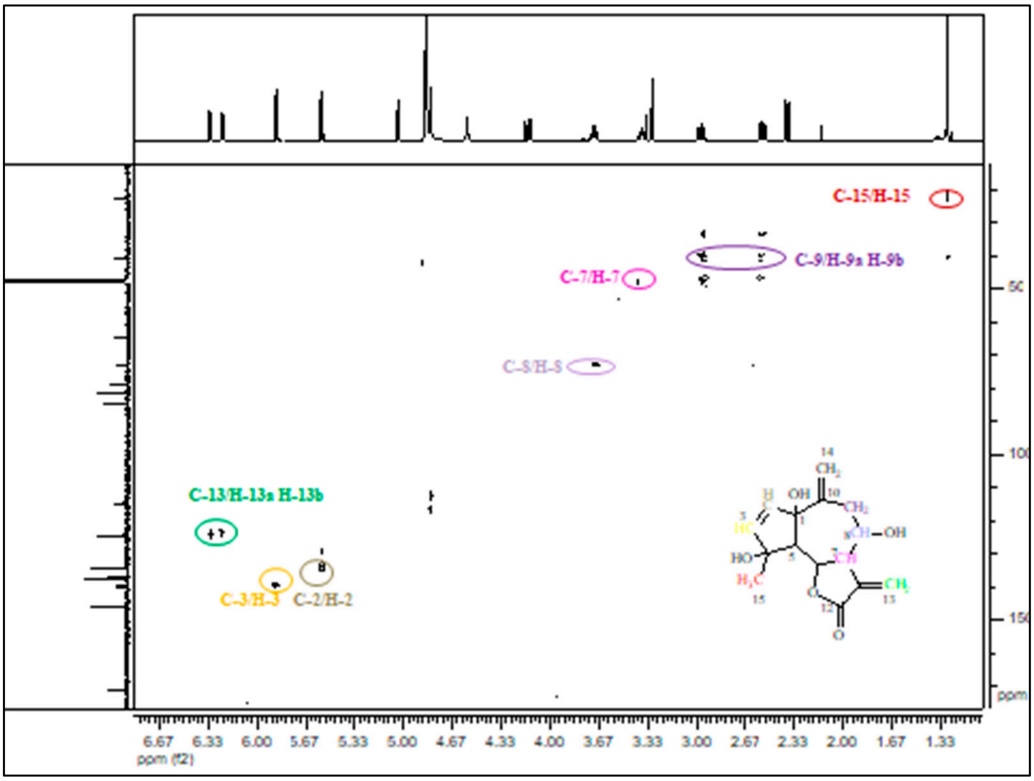

Figure S11. gHSQCAD spectrum of compound **3** (CD_3OD, 500 MHz).

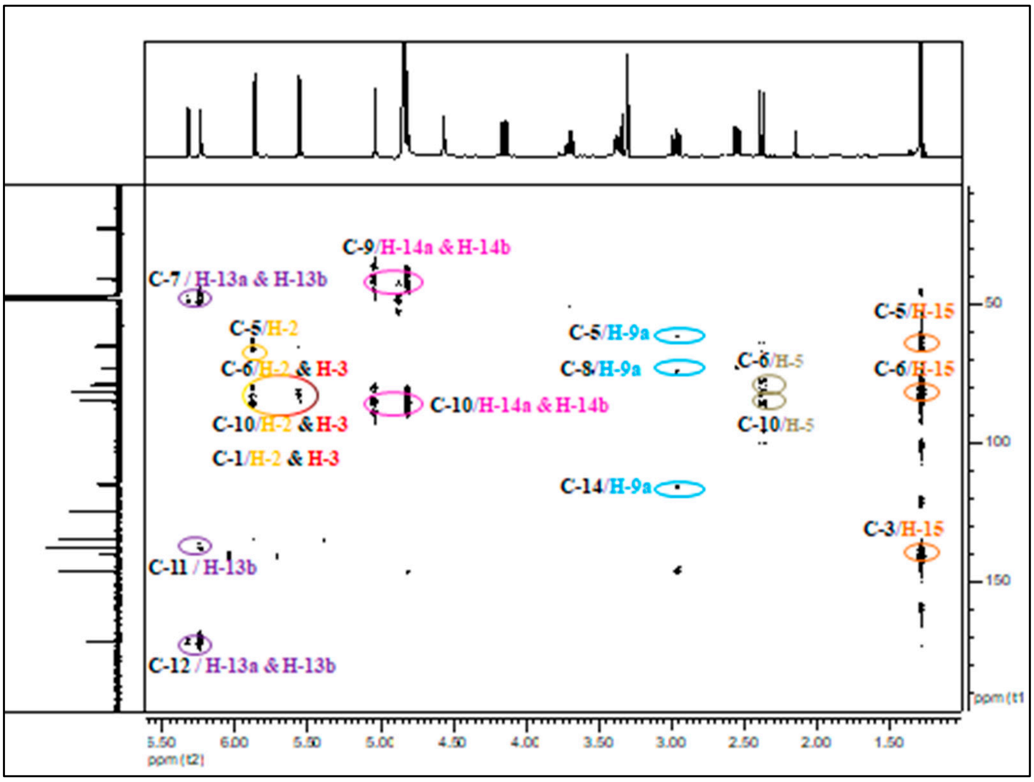

Figure S12. gHMBCAD spectrum of compound **3** (CD_3OD, 500 MHz).

Table S4. ^{1}H and ^{13}C NMR of compound **4** (CD_3OD, 500 MHz).

No	δC	Type C	δH	H	(Hz)
1	75.6	CH	3.59	1	dd (J = 10.0, 6.5)
2a	46.0	CH_2	2.58-2.66	1	o.s
2b			2.58-2.66	1	o.s
3	210.0	C=O	-	-	-
4	44.1	CH	2.58-2.66	1	o.s
5	49.5	CH	1.56-1.68	1	o.s
6	83.5	CH	4.09	1	t (J = 10.5)
7	50.3	CH	2.58-2.66	1	o.s
8a	20.8	CH_2	2.09-2.12	1	o.s
8b			1.56-1.68	1	o.s
9a	36.0	CH_2	2.09-2.12	1	o.s
9b			1.37	1	m
10	42.0	C	-	-	-
11	139.1	C	-	-	-
12	116.3	C=O	-	-	-
13a	171.1	CH_2	6.02	1	d (J = 3.0)
13b			5.51	1	d (J = 3.0)
14	12.6	CH_3	1.11	3	s
15	14.6	CH_3	1.21	3	d (J = 6.5)

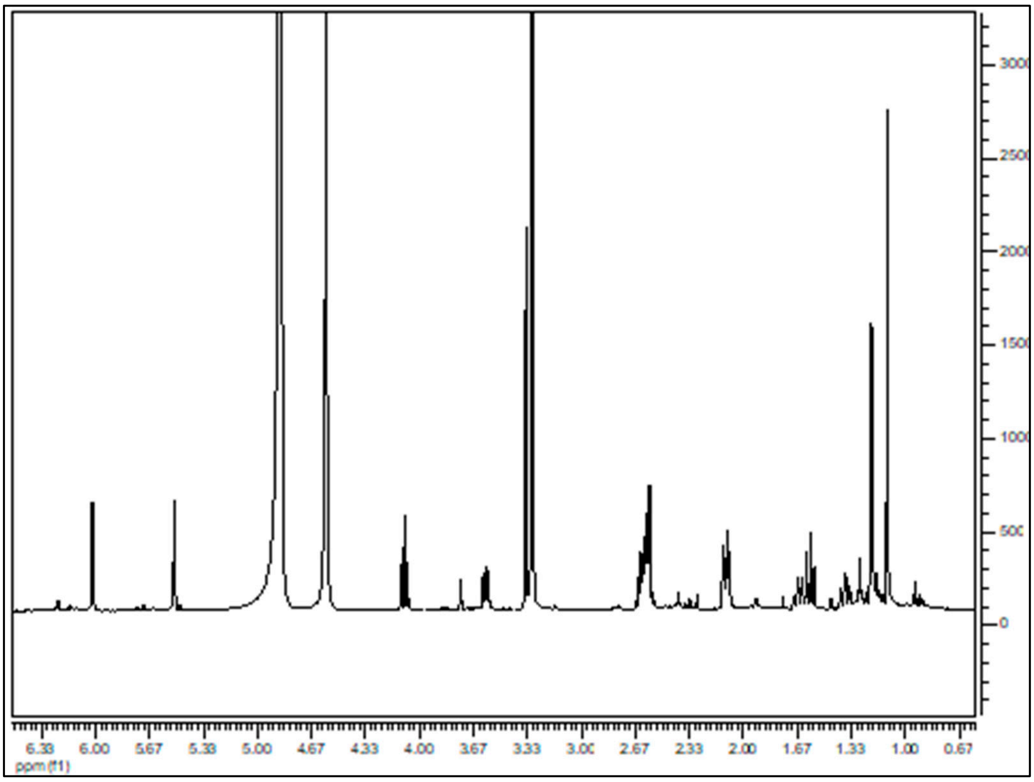

(A)

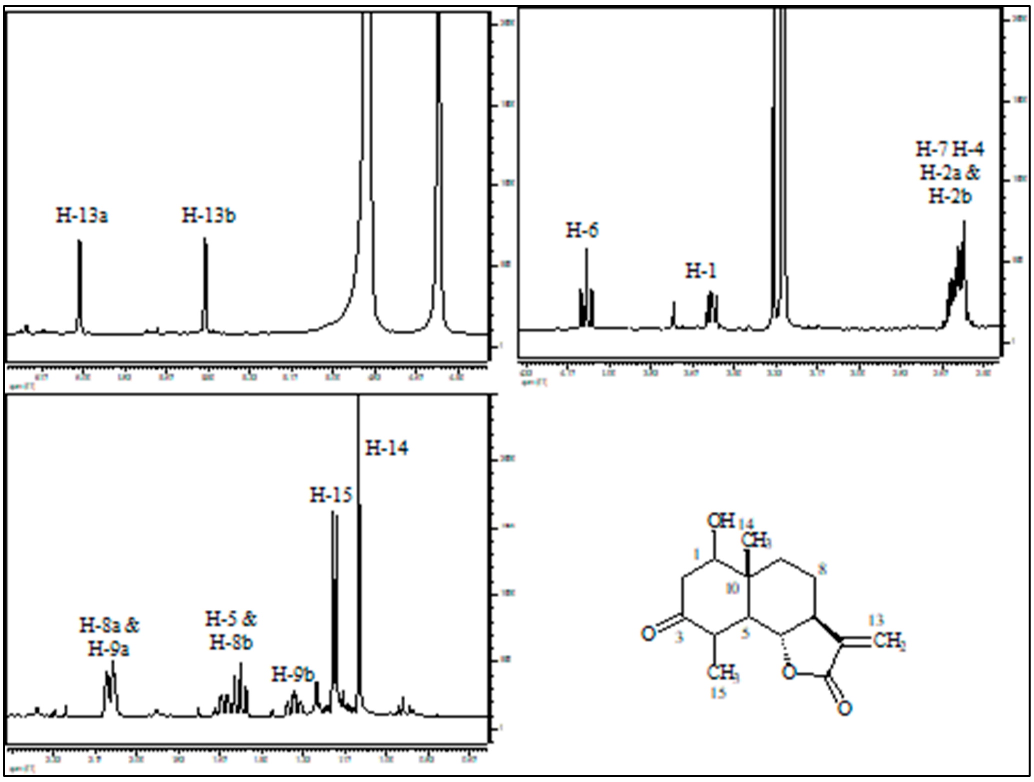

(B)

Figure S13. ^{1}H-NMR full spectrum (A) and parts thereof (B) of compound **4** (CD_3OD, 500MHz).

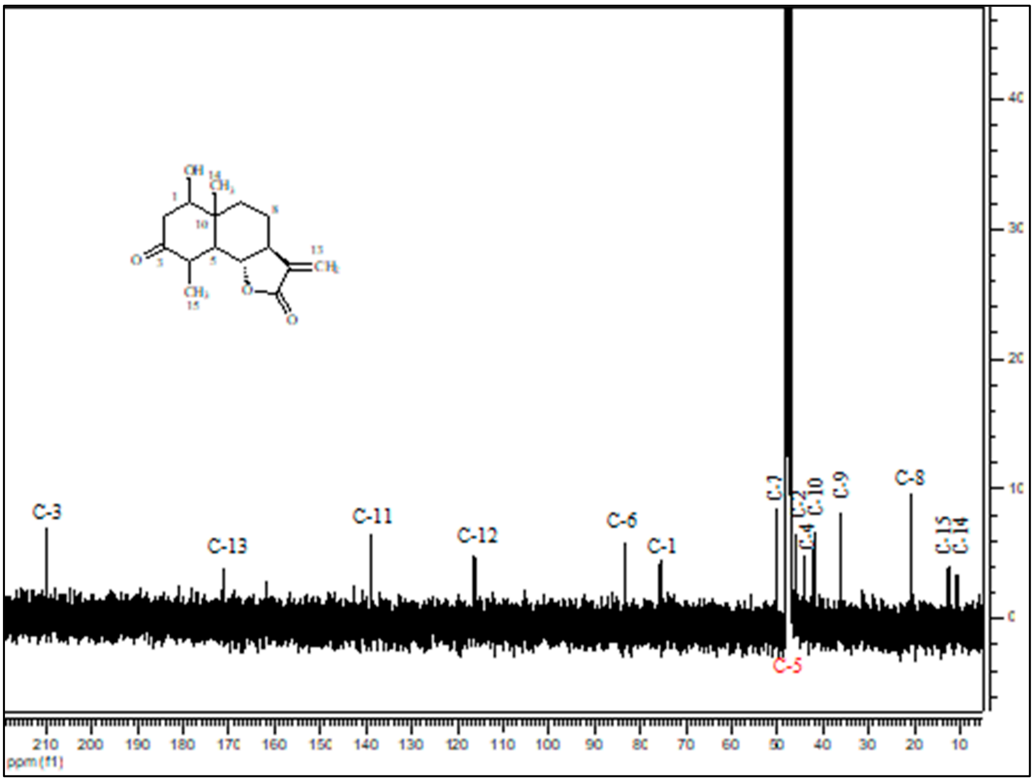

Figure S14. ^{13}C-NMR spectrum of compound **4** (CD_3OD, 125MHz).

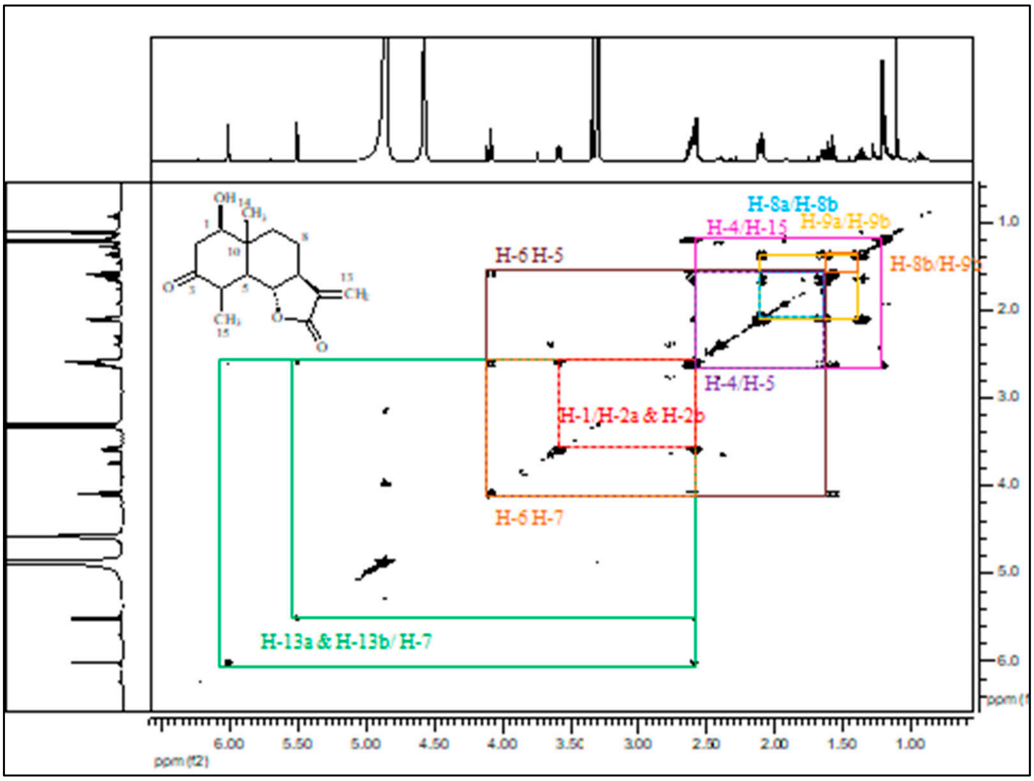

Figure S15. gDQCOSY spectrum of compound **4** (CD_3OD, 500MHz).

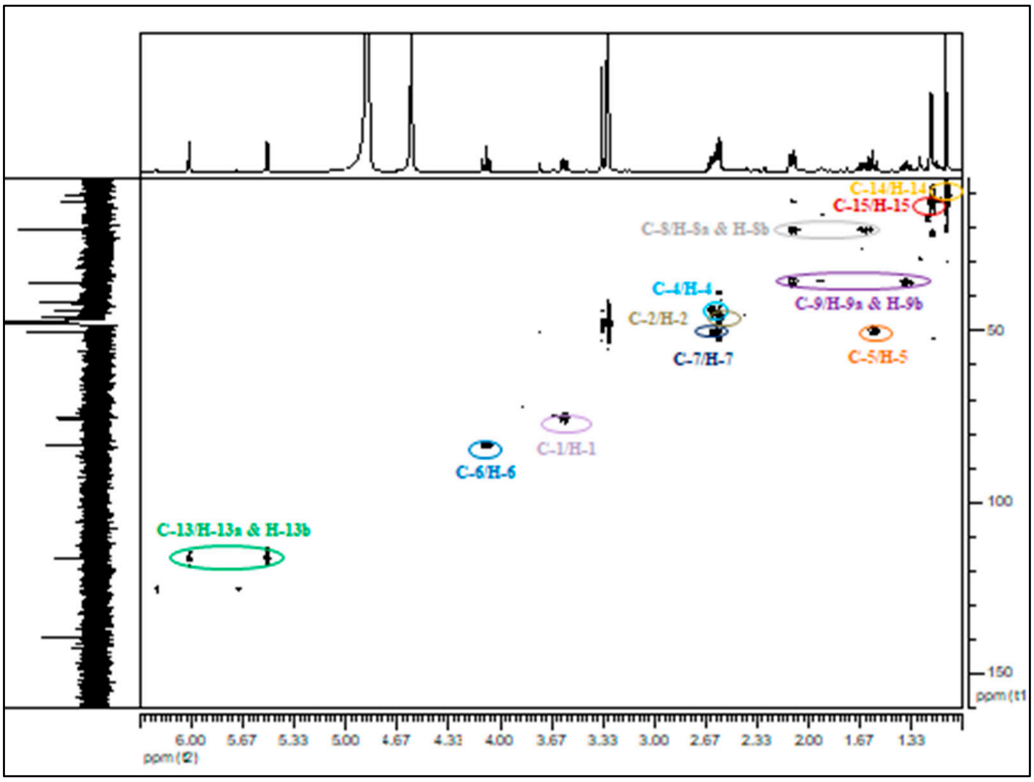

Figure S16. gHSQCAD spectrum of compound **4** (CD_3OD, 500MHz).

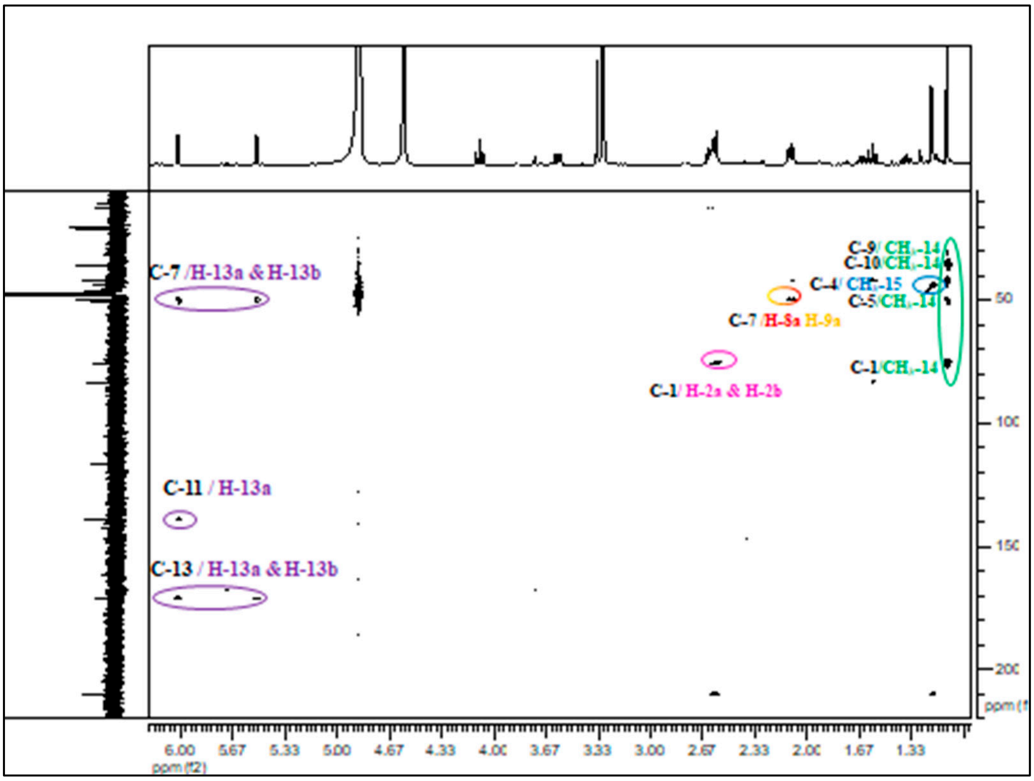

Figure S17. gHMBCAD spectrum of compound **4** (CD_3OD, 500MHz).

Table S5. ^{1}H and ^{13}C NMR of compound **5** (CD_3OD, 500 MHz).

No	δ_C	Type C	δ_H	H	J (Hz)
1	75.5	HO-CH	3.53	-	dd (J = 12.0, 5.5)
2a	39.8	CH_2	2.00–2.30	1	o.s
2b			1.67–1.33	1	o.s
3	69.1	HO-CH	4.02	1	m
4	146.1	C=	-	-	-
5	49.8	CH	1.51	-	m
6	79.7	O-CH	4.18	1	t (J = 10.5)
7	49.5	CH	2.61	1	m
8a	20.9	CH_2	2.00-2.30	1	o.s
8b			1.67–1.33		o.s
9a	35.6	CH_2	1.67–1.33	1	o.s
9b			1.67–1.33	1	o.s
10	42.7	C	-	-	-
11	139.7	C	-	-	-
12	171.4	C=O	-	-	-
13a	116.0	CH_2	6.01	1	d (J = 3.0)
13b			5.50	1	d (J = 3.0)
14	10.6	CH_3	0.75	3	s
15a	105.6	$=CH_2$	5.28	1	s
15b			4.93	1	s

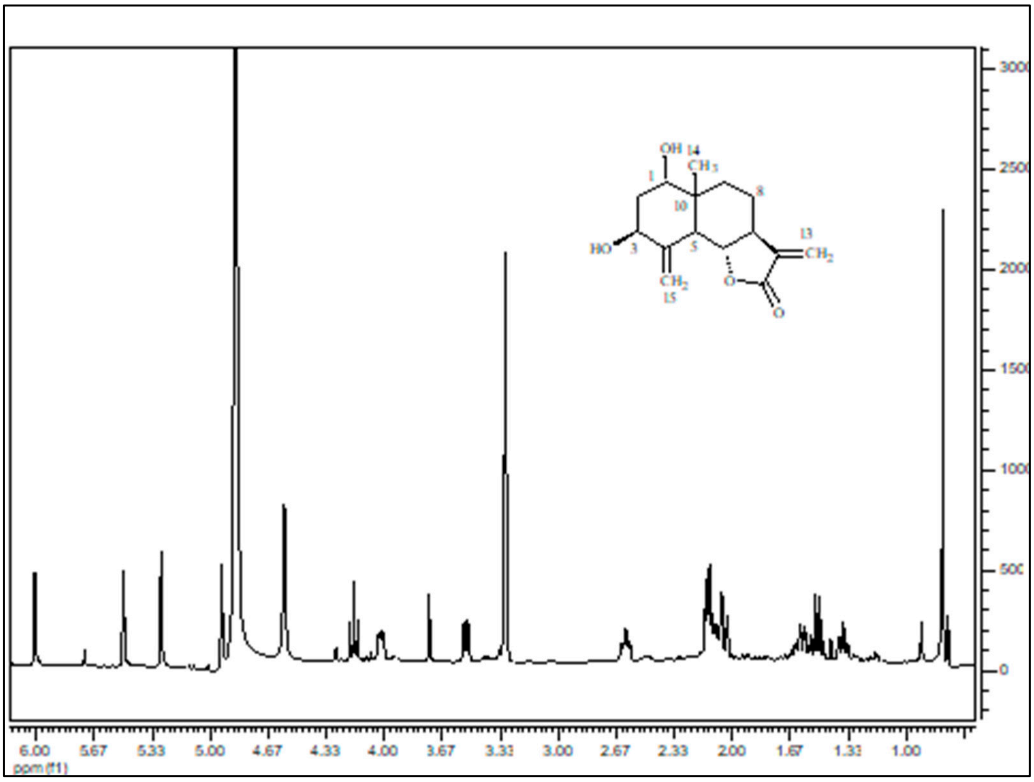

(A)

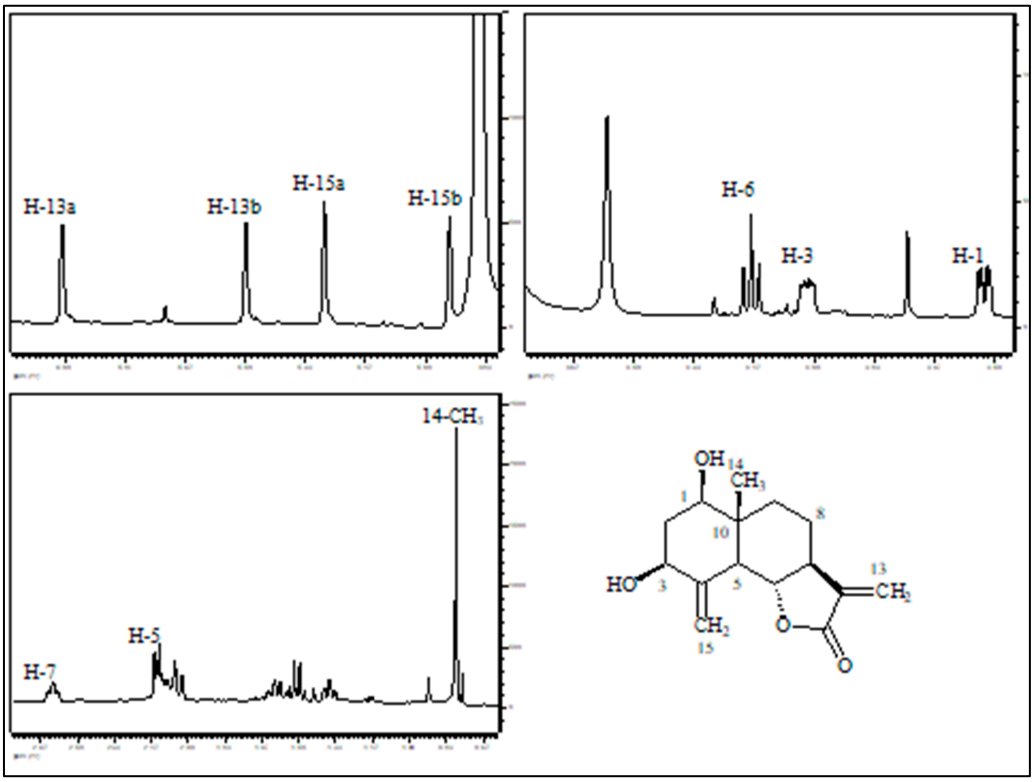

(B)

Figure S18. ^{1}H-NMR full spectrum (A) and parts thereof (B) of compound **5** (CD_3OD, 500MHz).

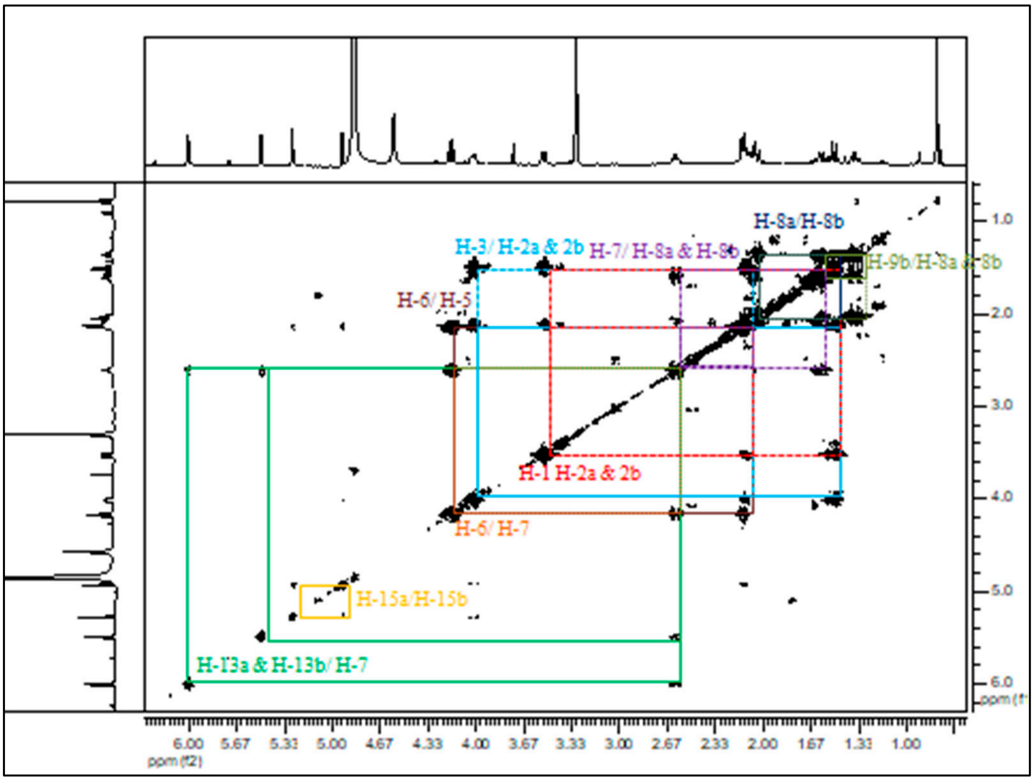

Figure S19. gDQCOSY spectrum of compound **5** (CD_3OD, 500MHz).

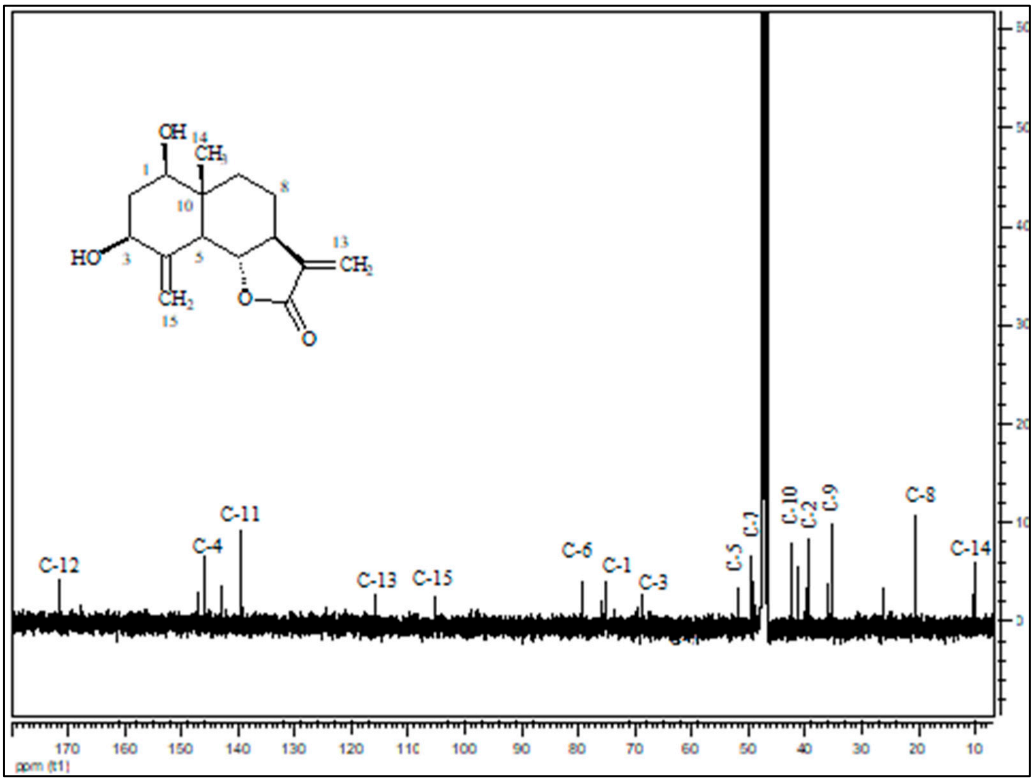

Figure S20. ^{13}C-NMR spectrum of compound **5** (CD_3OD, 125MHz).

Table S6. ^1H and ^{13}C NMR of compound **6** (CD_3OD, 500 MHz).

No	δ_C	Type C	δ_H	H	J (Hz)
1	76.1	HO-CH	3.42	1	dd (J = 12.0, 4.5)
2a	40.3	CH_2	2.07	1	o.s
2b			1.48	1	o.s
3	70.5	HO-CH	3.93	1	m
4	147.1	C=	-	-	-
5	52.1	CH	1.49	1	d (J = 8.5)
6	67.7	O-CH	4.08	1	t (J = 10.0)
7	50.8	CH	2.50	1	m
8a	26.6	CH_2	1.74	1	o.s
8b			1.62	1	o.s
9a	36.2	CH_2	1.90	1	o.s
9b			1.16	1	o.s
10	41.5	C	-	-	-
11	142.5	C	-	-	-
12	167.9	C=O	-	-	-
13a	124.7	CH_2	6.24	1	s
13b			5.72	1	s
14	10.6	CH_3	0.76	3	s
15a	104.5	$=CH_2$	5.28	1	s
15b			4.80	1	s
$-OCH_3$	49.2	$-OCH_3$	3.74	3	s

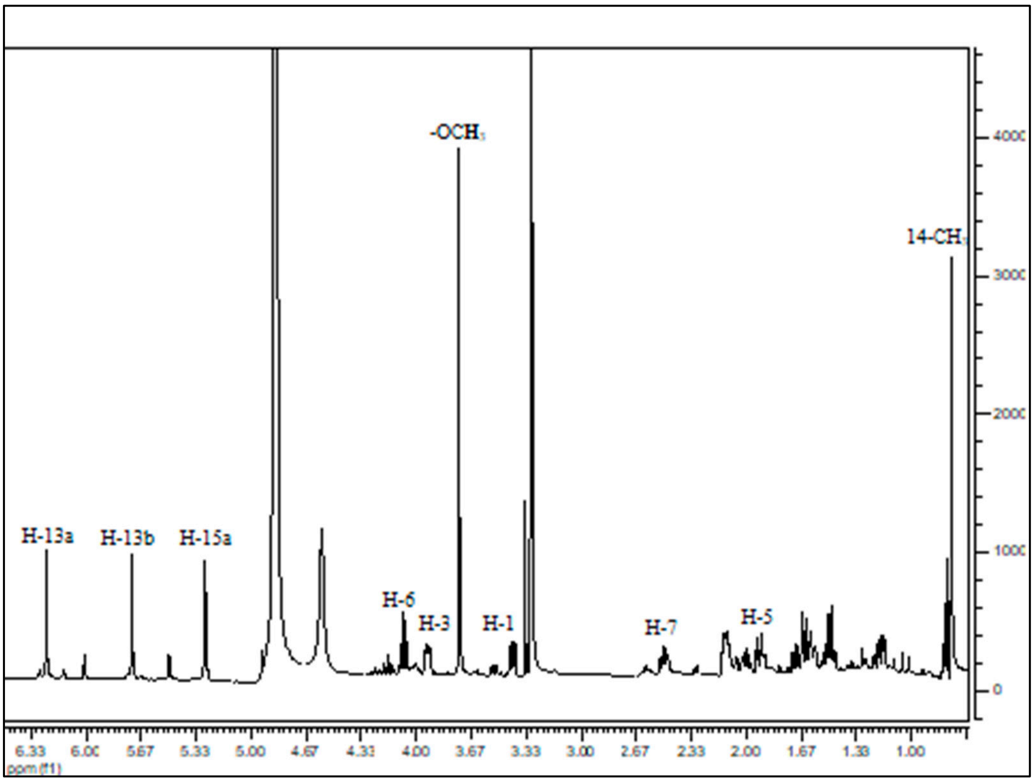

(A)

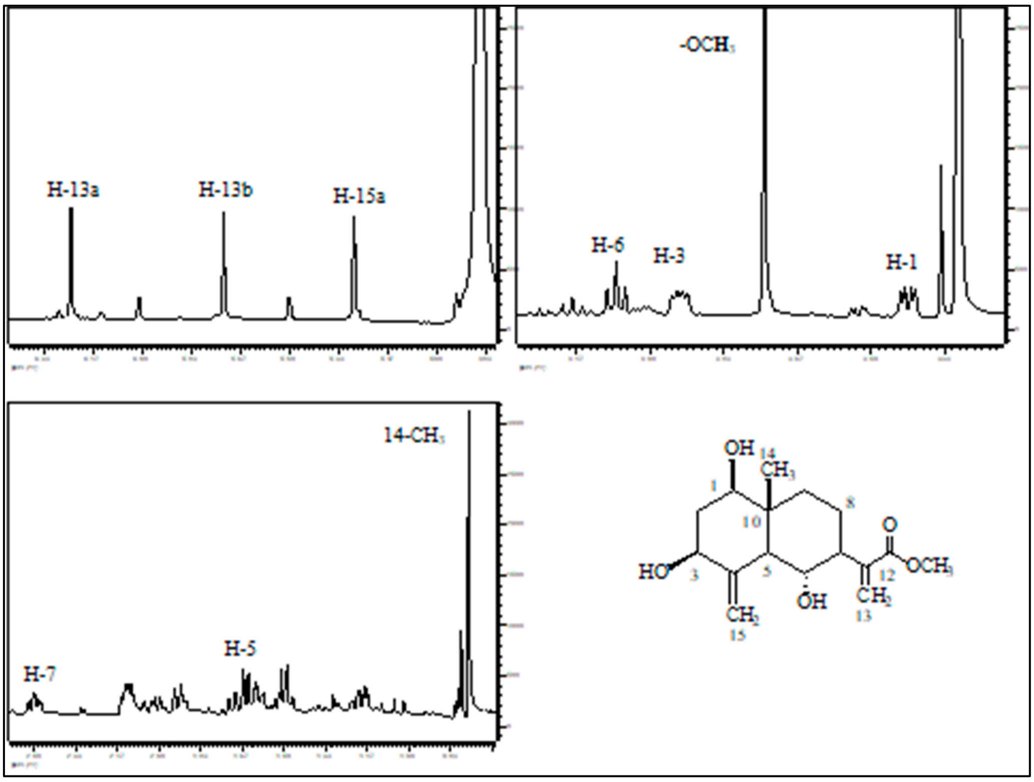

(B)

Figure S21. ^{1}H-NMR full spectrum (A) and parts thereof (B) of compound **6** (CD_3OD, 500MHz).

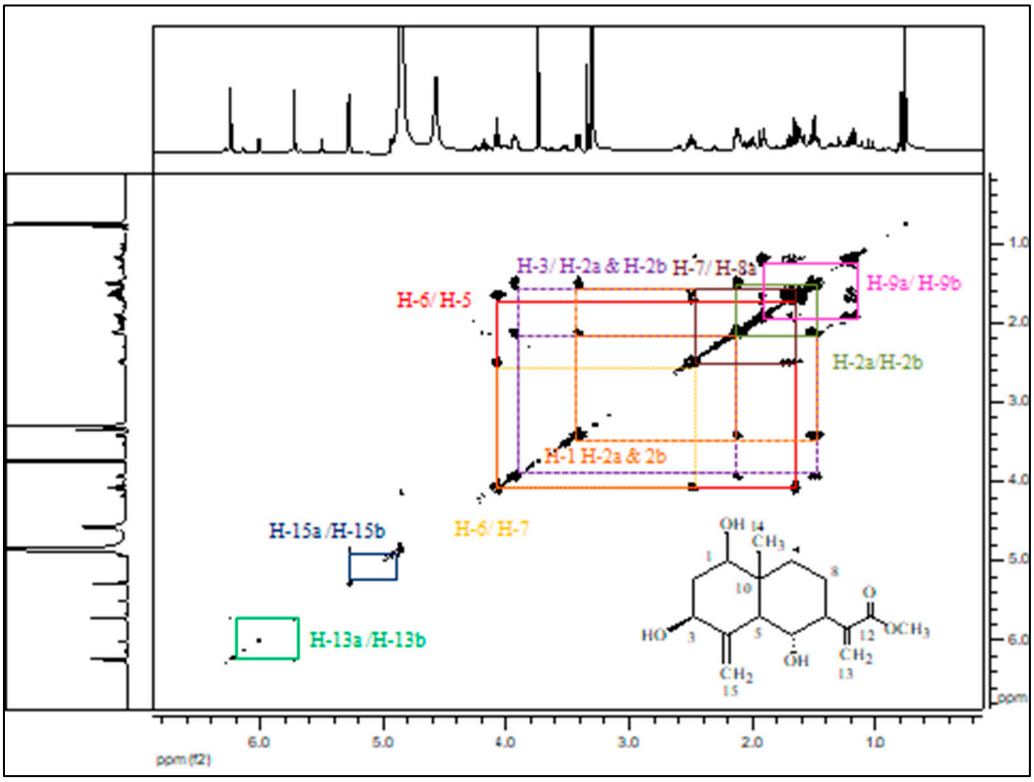

Figure S22. gDQCOSY spectrum of compound **6** (CD_3OD, 500MHz).

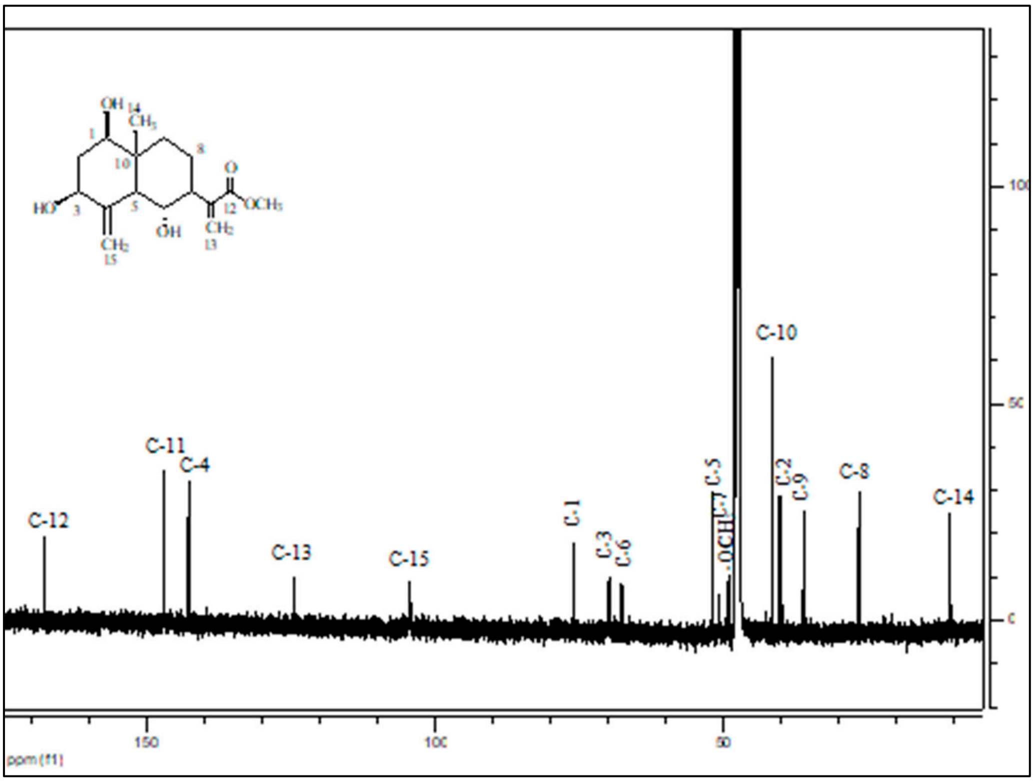

Figure S23. ^{13}C-NMR spectrum of compound **6** (CD_3OD, 125MHz).

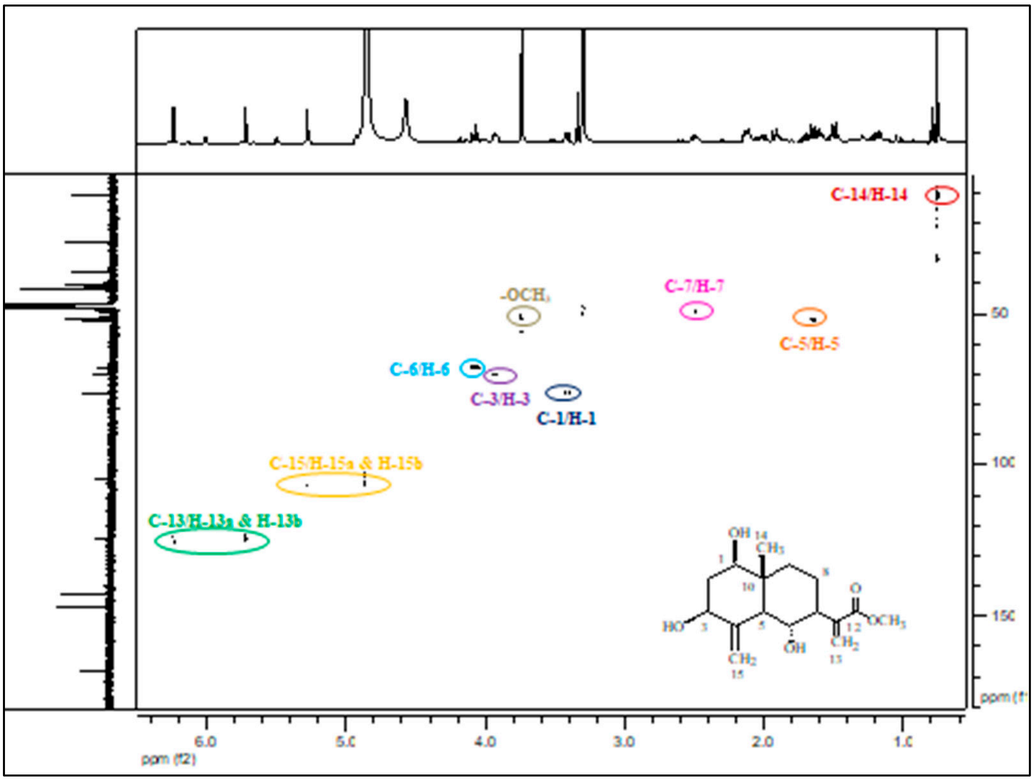

Figure S24. gHSQCAD spectrum of compound **6** (CD_3OD, 500MHz).

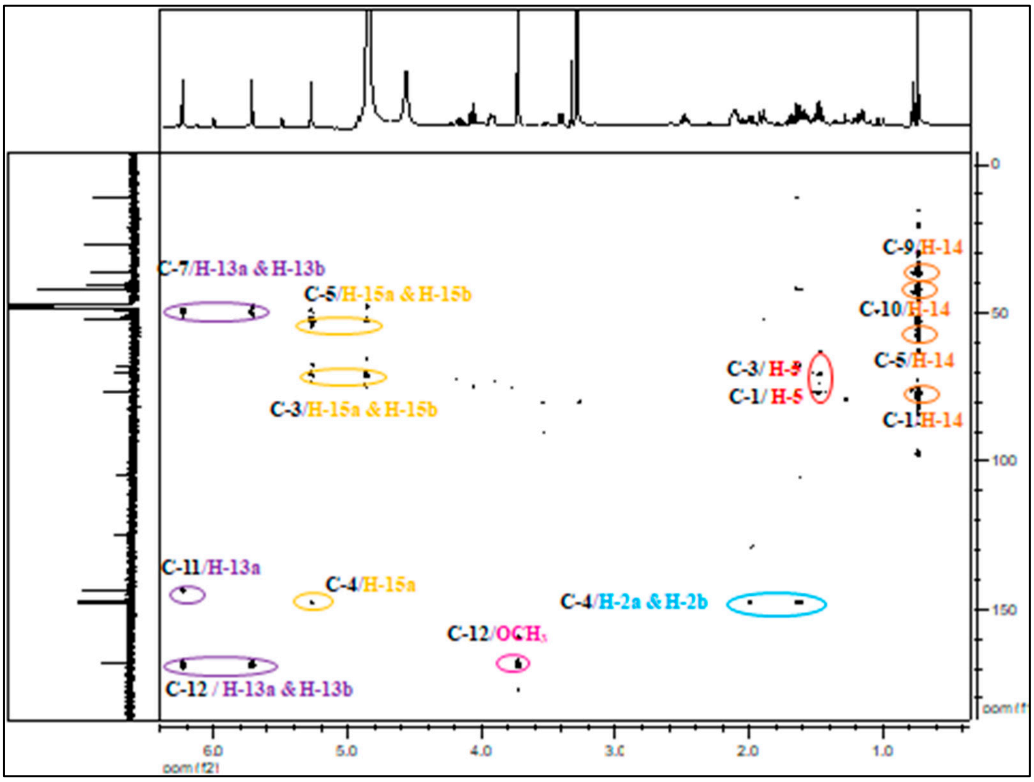

Figure S25. gHMBCAD spectrum of compound **6** (CD_3OD, 500MHz).

Table S7. ^{1}H and ^{13}C NMR of compound **7** (CD_3OD, 500 MHz).

No	δC	Type C	δH	H	J (Hz)
1	78.2	HO-CH	3.35	1	o.s
2a	27.3	CH_2	1.56–1.73	1	o.s
2b			1.56–1.73	1	o.s
3a	39.4	CH_2	1.56–1.73	1	o.s
3b			1.56–1.73	1	o.s
4	70.7	C	-	-	-
5	55.5	CH	1.37	1	d (J = 8.5)
6	72.8	HO-CH	4.29	1	t (J = 10.5)
7	49.8	CH	2.52	1	m
8a	26.8	CH_2	1.56–1.73	1	o.s
8b			1.56–1.73	1	o.s
9a	39.6	CH_2	1.86	1	m
9b			1.16	1	m
10	40.4	C	-	-	-
11	142.1	C	-	-	-
12	167.6	C=O	-	-	-
13a	125.4	CH_2	6.25	1	s
13b			5.73	1	s
14	13.0	CH_3	0.95	3	s
15	22.6	CH_3	1.32	3	s
-OCH_3	50.8	-OCH_3	3.75	3	s

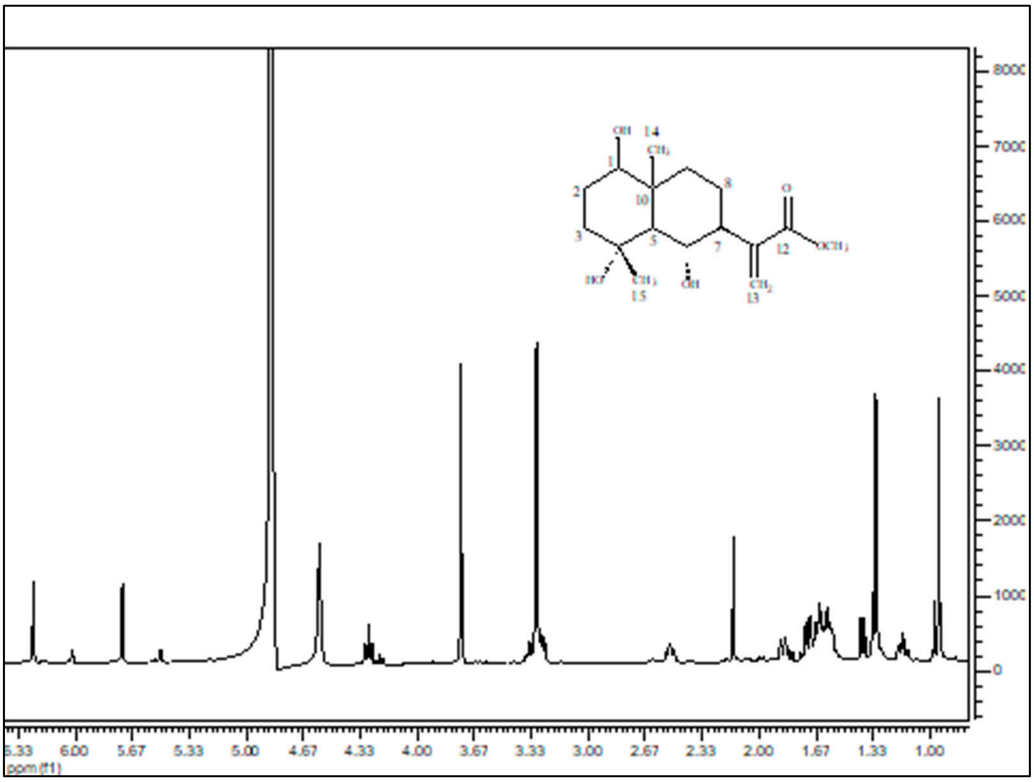

(A)

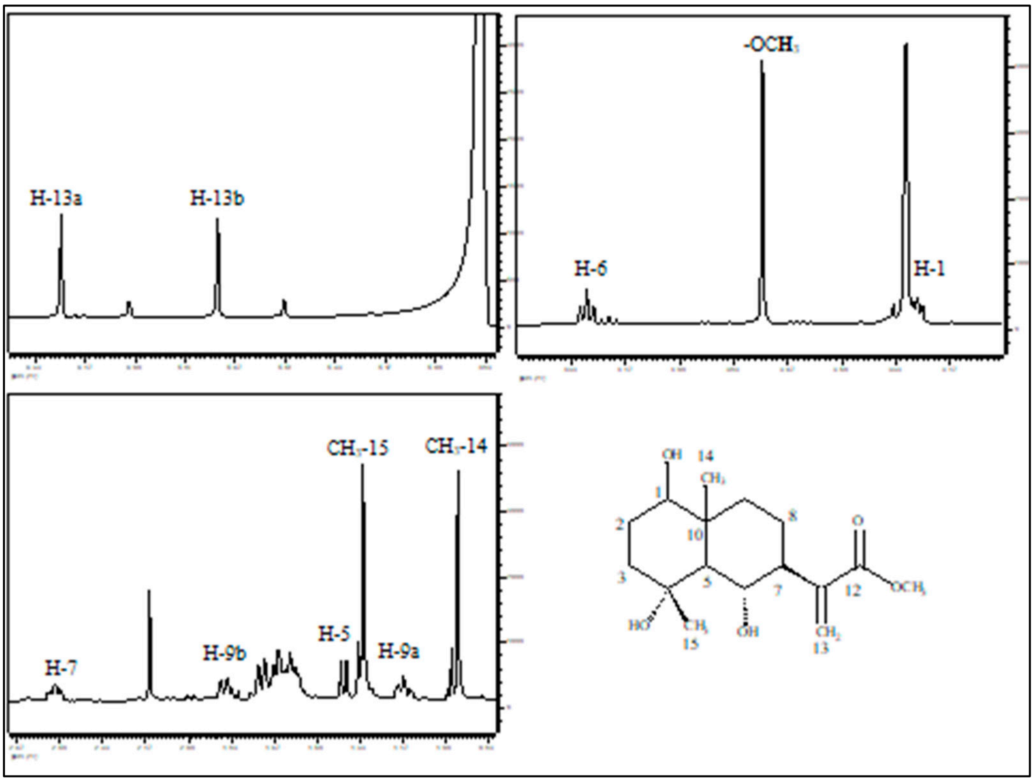

(B)

Figure S26. ^{1}H-NMR full spectrum (A) and parts thereof (B) of compound **7** (CD_3OD, 500MHz).

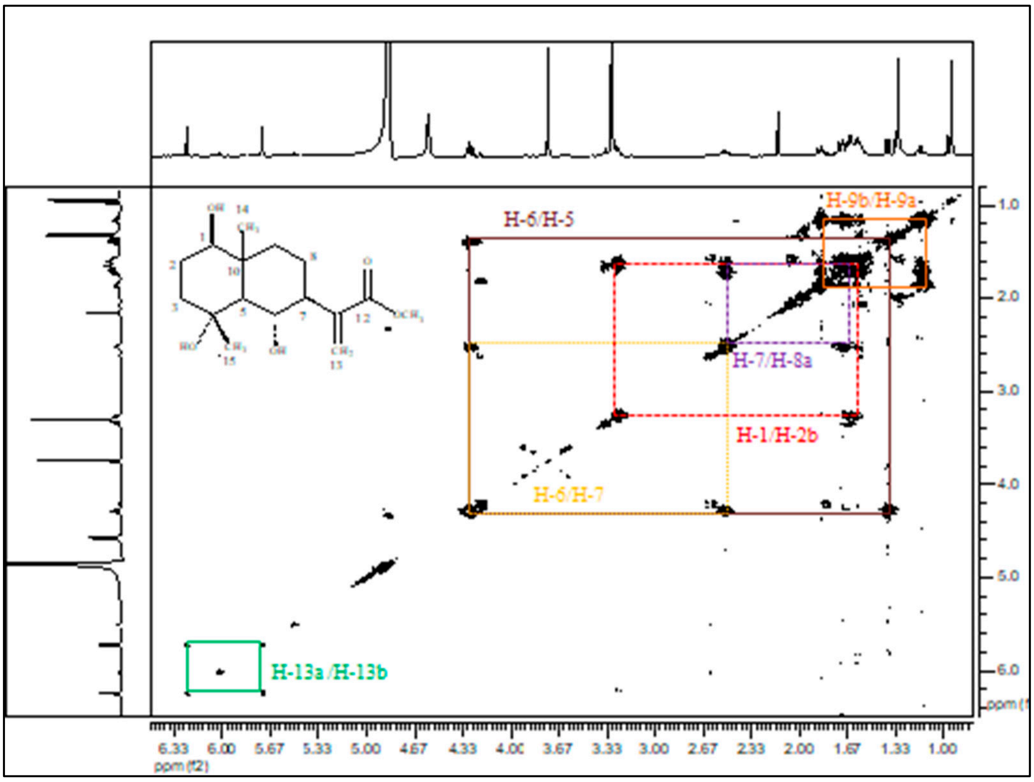

Figure S27. gDQCOSY spectrum of compound 7 (CD_3OD, 500MHz).

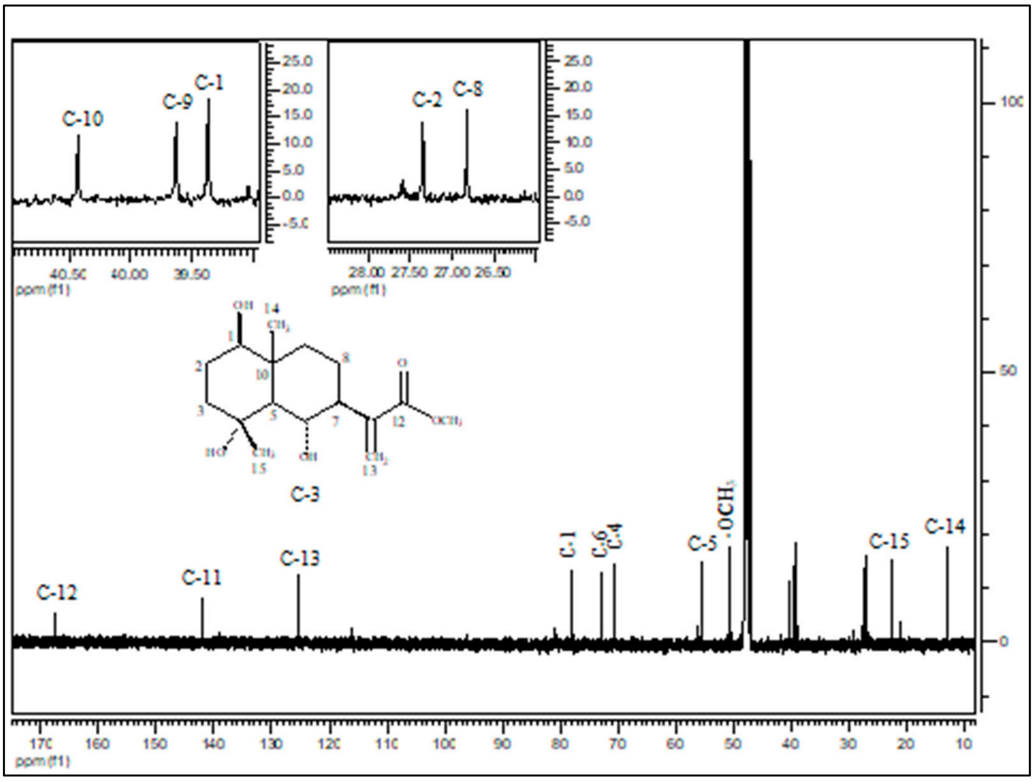

Figure S28. ^{13}C-NMR spectrum of compound **7** (CD_3OD, 125MHz).

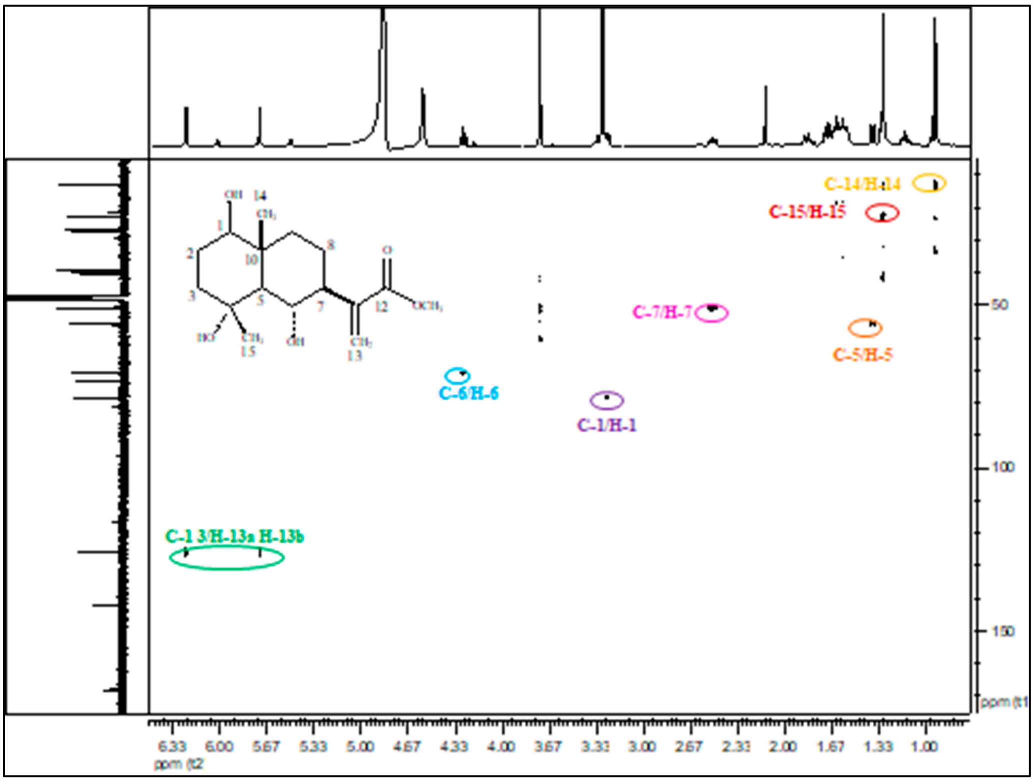

Figure S29. gHSQCAD spectrum of compound **7** (CD_3OD, 500MHz).

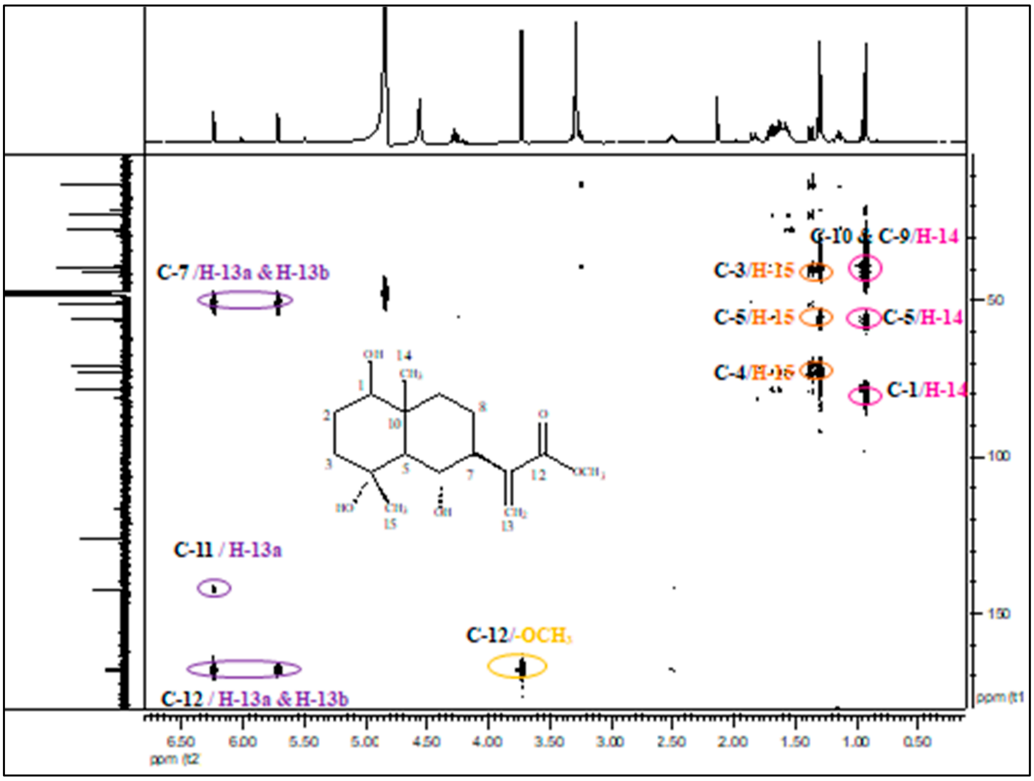

Figure S30. gHMBCAD spectrum of compound **7** (CD_3OD, 500MHz).

Table S8. ^{1}H and ^{13}C NMR of compound **8** (CD_3OD, 500 MHz).

No	δC	Type C	δH	H	J (Hz)
1	76.9	HO-C	-	-	-
2	59.8	CH	3.46	1	d (J = 3.0)
3	56.6	CH	3.60	1	d (J = 3.0)
4	76.8	C	-	-	-
5	156.4	C	-	-	-
6	117.2	CH	6.78	1	s
7	154.5	-	-	-	-
8	76.5	HO-CH	5.59	1	d (J = 12.0)
9	60.9	CH	2.73	1	d (J = 12.0)
10	206.4	C=O	-	-	-
11	120.5	C	-	-	-
12	174.5	C=O	-	-	-
13	7	CH_3	1.90	3	s
14	29.9	CH_3	2.42	3	s
15	23.3	CH_3	1.48	3	s

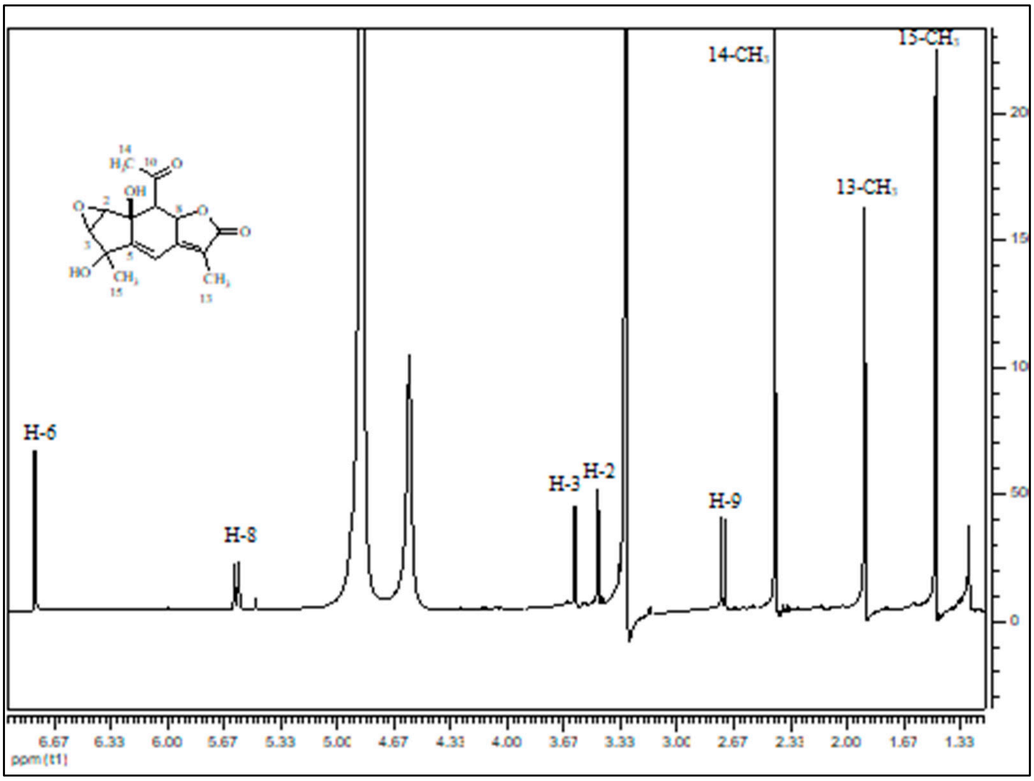

Figure S31. ^{1}H-NMR spectrum of compound **8** (CD_3OD, 500MHz).

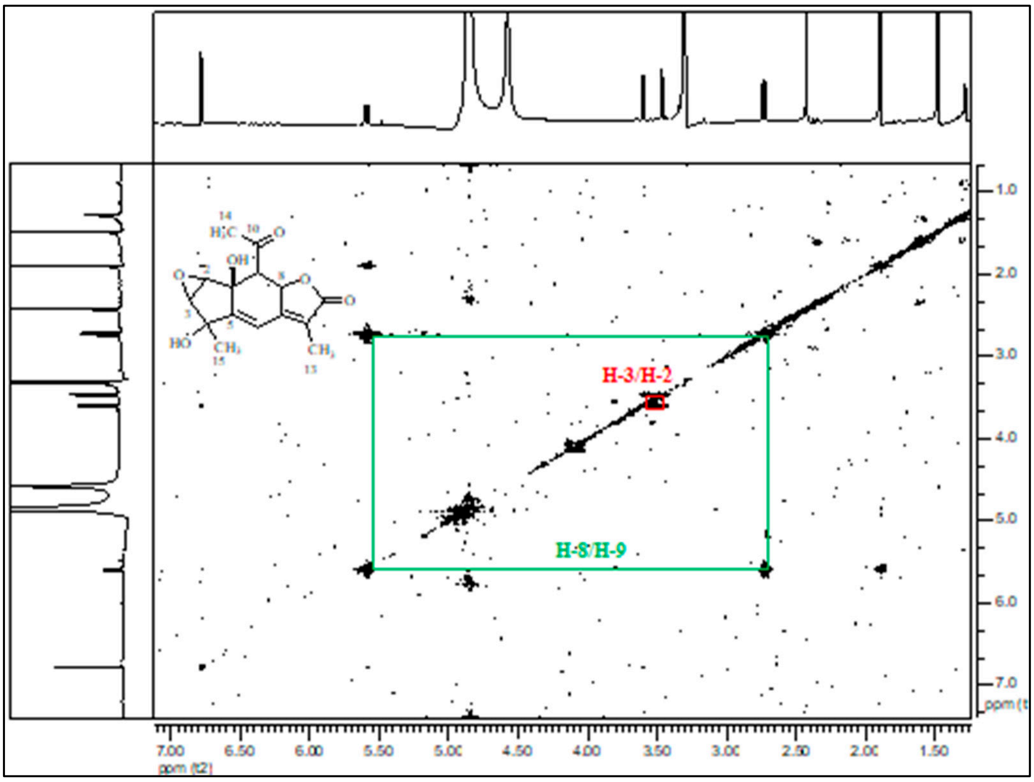

Figure S32. gDQCOSY spectrum of compound **8** (CD_3OD, 500MHz).

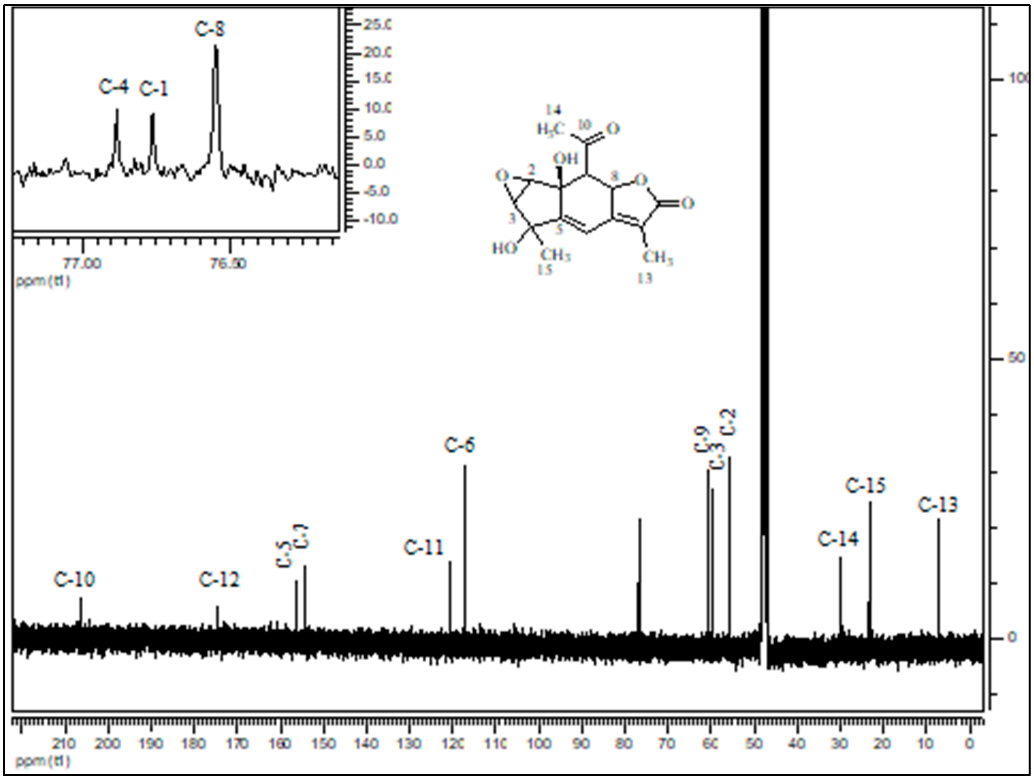

Figure S33. ^{13}C-NMR spectrum of compound **8** (CD_3OD, 125MHz).

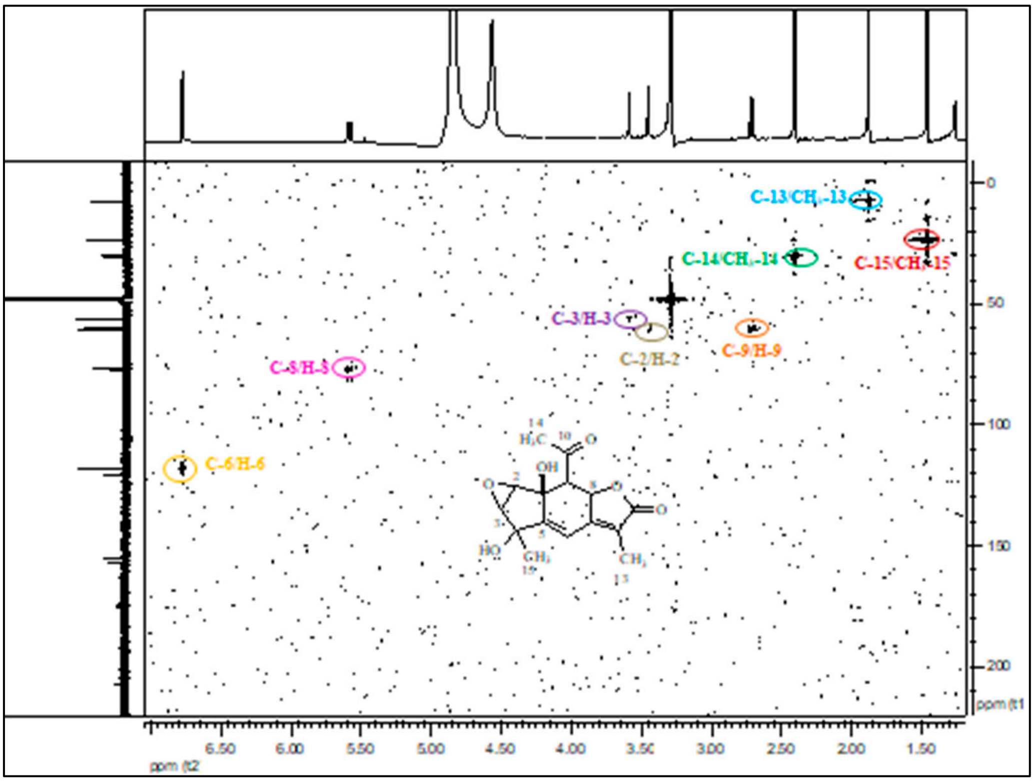

Figure S34. gHSQCAD spectrum of compound **8** (CD_3OD, 500MHz).

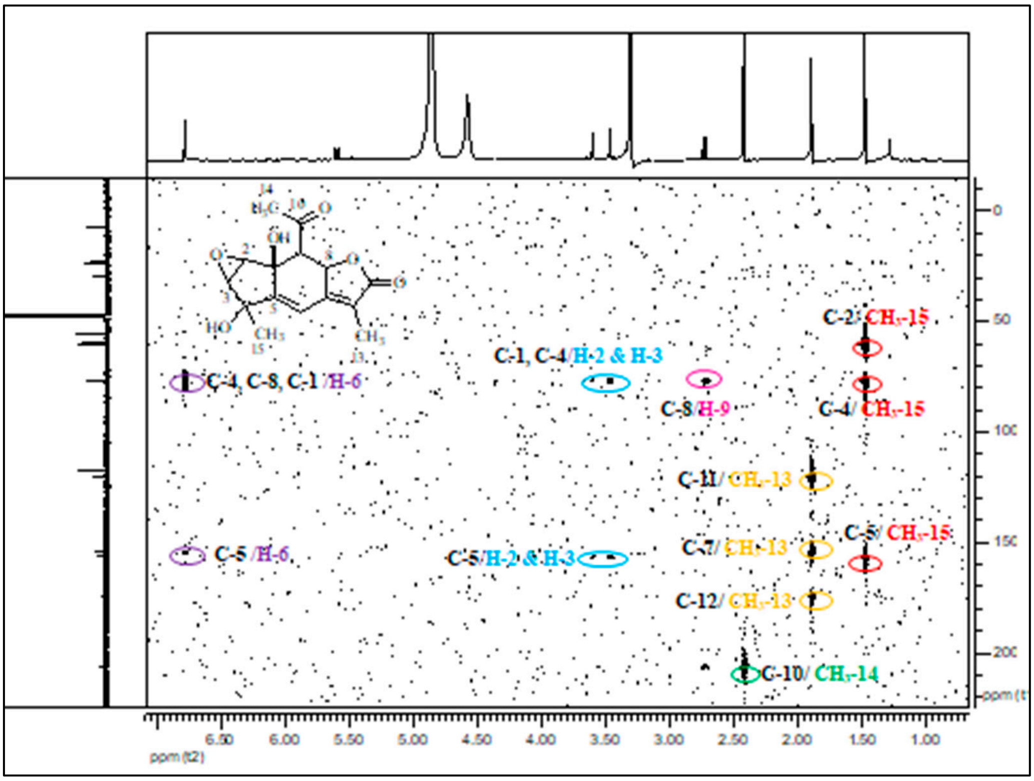

Figure S35. gHMBCAD spectrum of compound **8** (CD_3OD, 500MHz).

Table S9. ^{1}H and ^{13}C NMR of compound **9** (CD_3OD, 500 MHz).

No	δC	C	δH	H	J (Hz)
1	208.7	C=O	-	-	-
2	34.0	CH_2	2.36	2	br d (J = 5.0)
3	31.9	CH_2	2.64	2	br t (J = 4.0)
4	171.2	C	-	-	-
5	136.1	C	-	-	-
6	73.6	CH	5.31	1	d (J = 4.0)
7	48.5	CH	3.18	1	br s
8	68.4	HO-CH	4.27	1	m
9a	45.9	CH_2	2.73	1	dd (J = 16.5, 9.5)
9b			2.57	1	dd (J = 16.5, 3.0)
10	208.0	C=O	-	-	-
11	136.3	C	-	-	-
12	176.6	C=O	-	-	-
13a	122.8	CH_2	6.30	1	o.s
13b			5.80	1	o.s
14	29.4	CH_3	2.20	1	s
15	15.9	CH_3	2.18	3	s

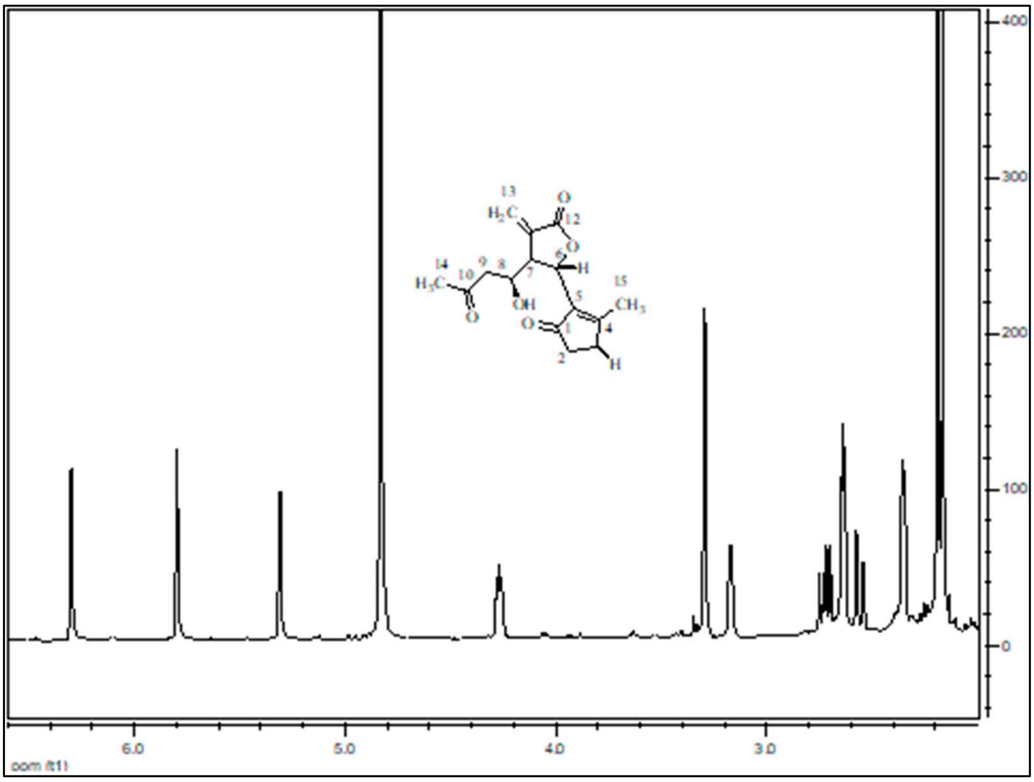

(A)

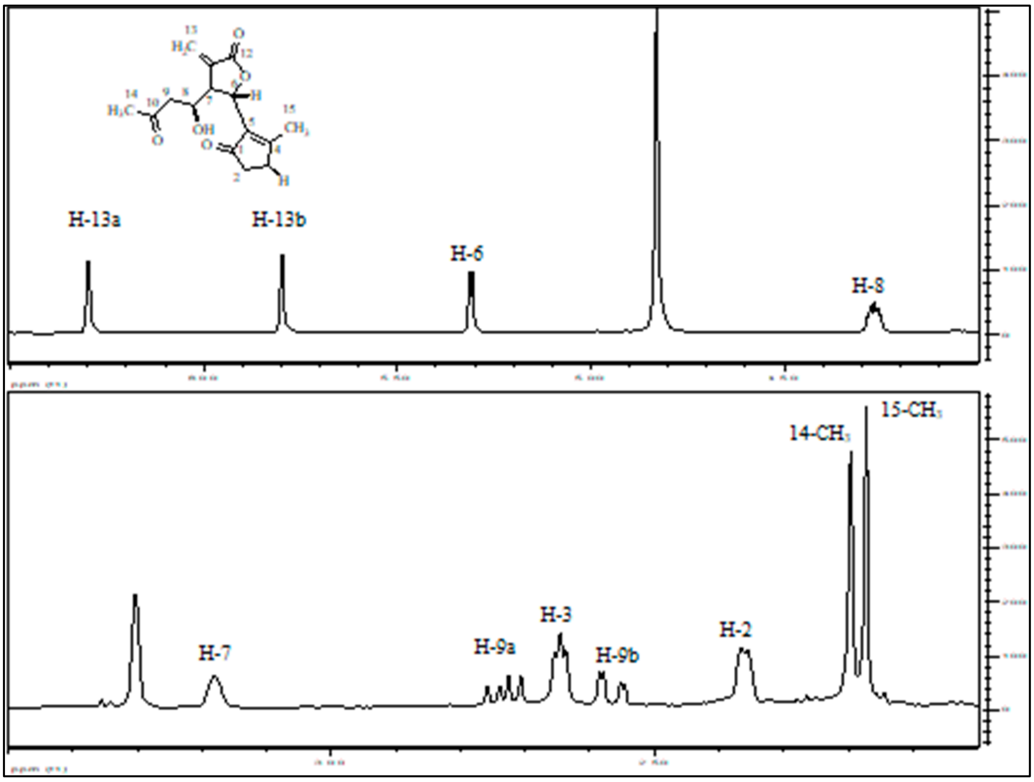

(B)

Figure S36. ^{1}H-NMR full spectrum (A) and parts thereof (B) of compound **9** (CD_3OD, 500MHz).

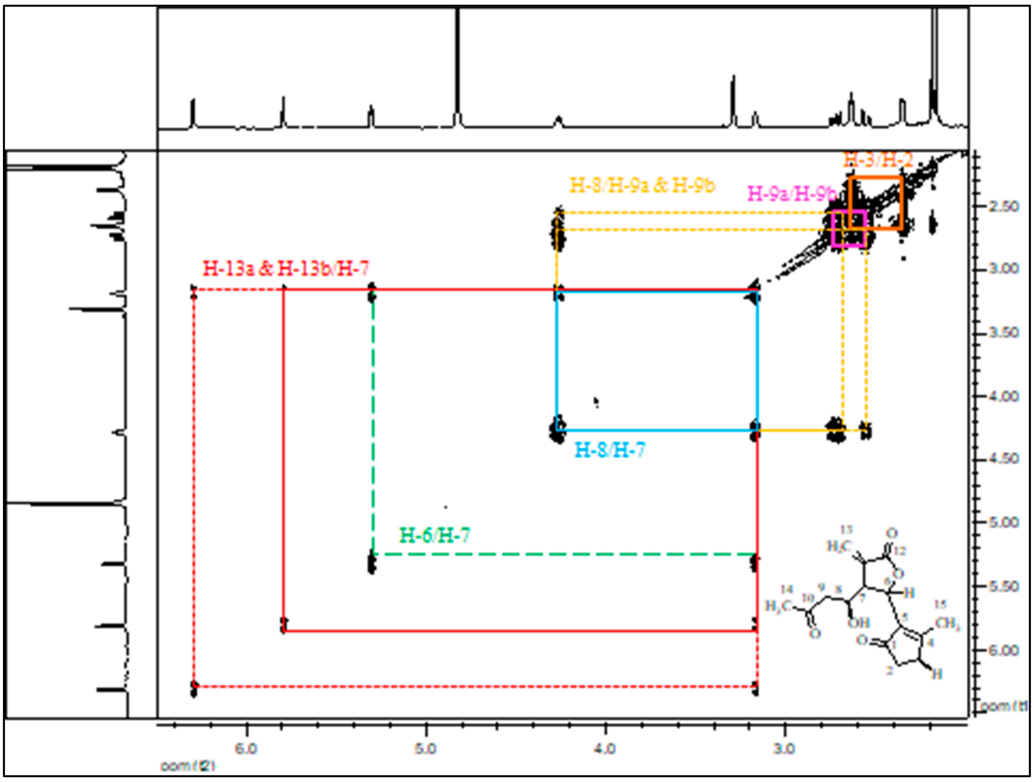

Figure S37. gDQCOSY spectrum of compound **9** (CD_3OD, 500MHz).

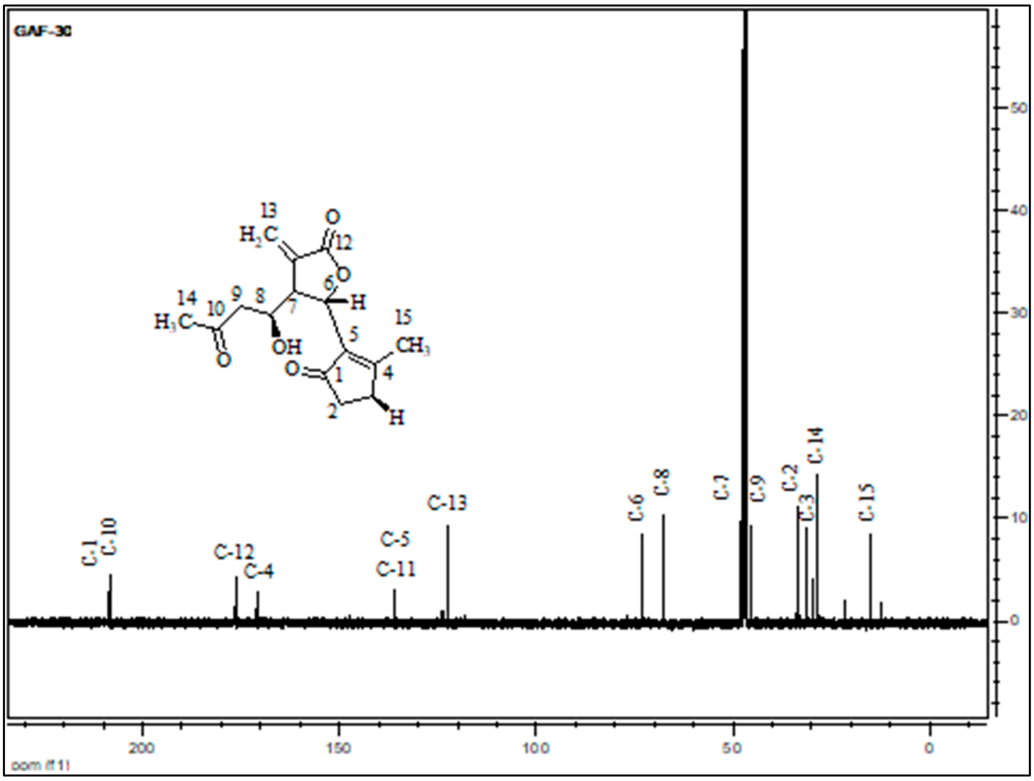

Figure S38. ^{13}C-NMR spectrum of compound **9** (CD_3OD, 125MHz).

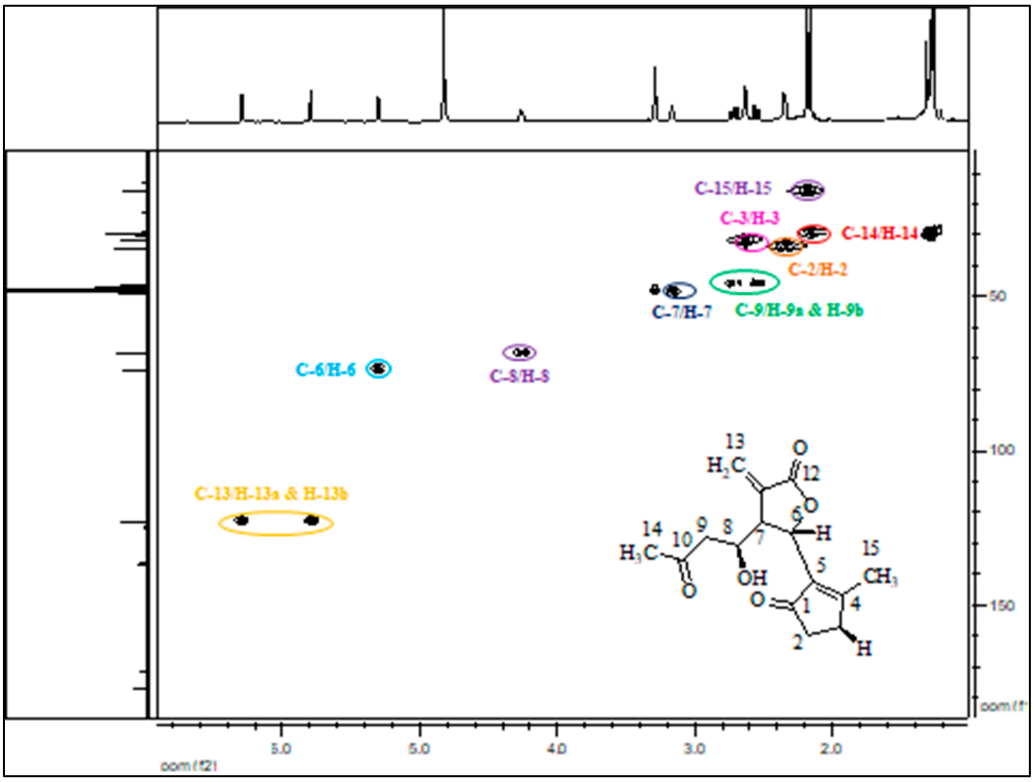

Figure S39. gHSQCAD spectrum of compound **9** (CD_3OD, 500MHz).

Table S10. ^{1}H and ^{13}C NMR of compound **10** (CD_3OD, 500 MHz).

No	Type C	δH	H	J (Hz)
1	HO-C	-	-	-
2a	CH	2.38	1	o.s
2b		1.95	1	dd (J = 13.5, 8.0)
3	HO-CH	3.78	1	d (J = 8.0)
4	C	-	-	-
5	C	-	-	-
6	CH	6.74	1	s
7	C	-	-	-
8	CH	5.57	1	d (J = 12.0)
9	CH	2.61	1	d (J = 12.0)
10	C=O	-	-	-
11	C	-	-	-
12	C=O	-	-	-
13	CH_3	1.90	3	s
14	CH_3	2.36	3	s
15	CH_3	1.52	3	s
-OCH_3	CH_3	3.44	3	s

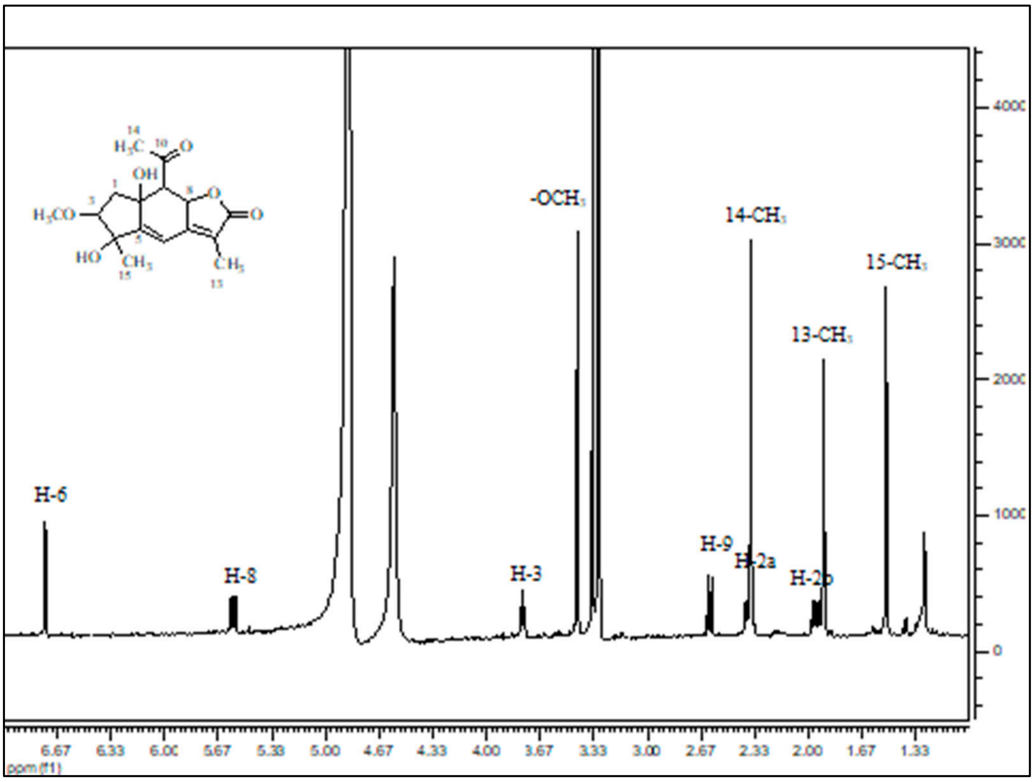

Figure S40. ^{1}H-NMR spectrum of compound **10** (CD_3OD, 500MHz).

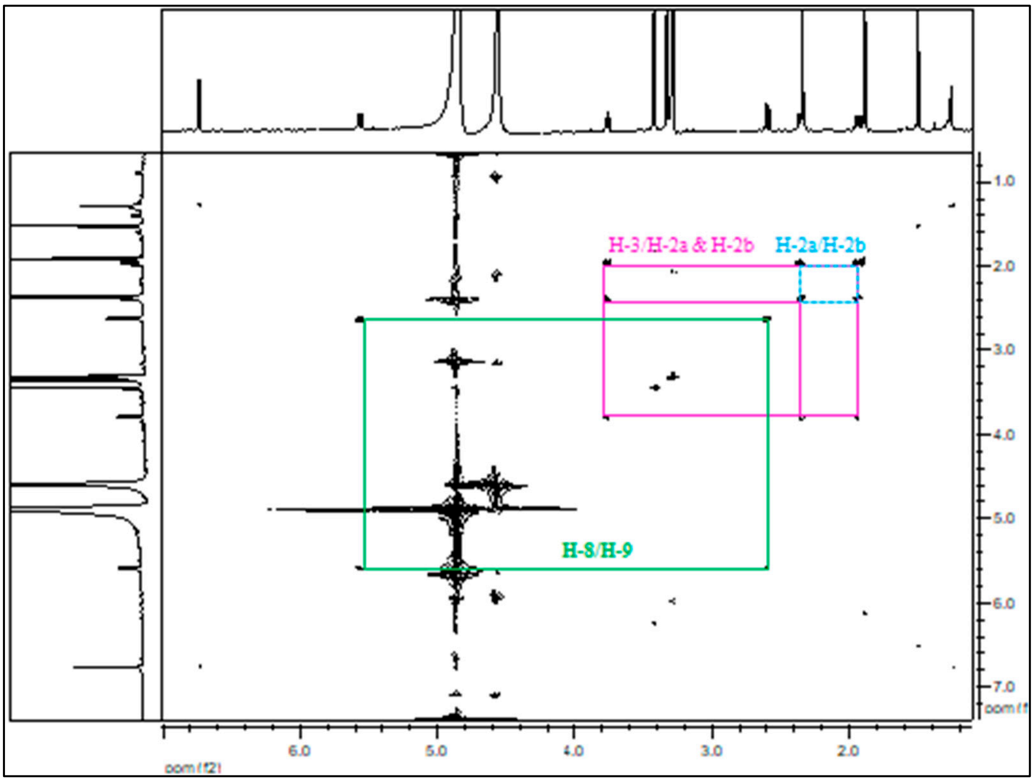

Figure S41. gDQCOSY spectrum of compound **10** (CD_3OD, 500MHz).

Table S11. ^1H and ^{13}C NMR of compound **11** (CD_3OD, 500MHz).

No	δ_C	C	δ_H	H	J (Hz)
1	35.8	C	-	-	-
2a	46.5	CH_2	1.52	1	dd (J = 14.5, 2.5)
2b			1.99	1	dd (J = 14.5, 2.5)
3	65.8	CH-OH	4.20	1	m
4a	45.0	CH_2	1.71	1	o.s
4b			2.42	1	dd (J = 13.5, 2.5)
5	87.6	C	-	-	-
6	173.0	C=	-	-	-
7	111.9	CH	5.74	1	s
8	184.3	C=O	-	-	-
9	26.0	CH_3	1.46	3	s
10	29.6	CH_3	1.27	3	s
11	25.5	CH_3	1.76	3	s

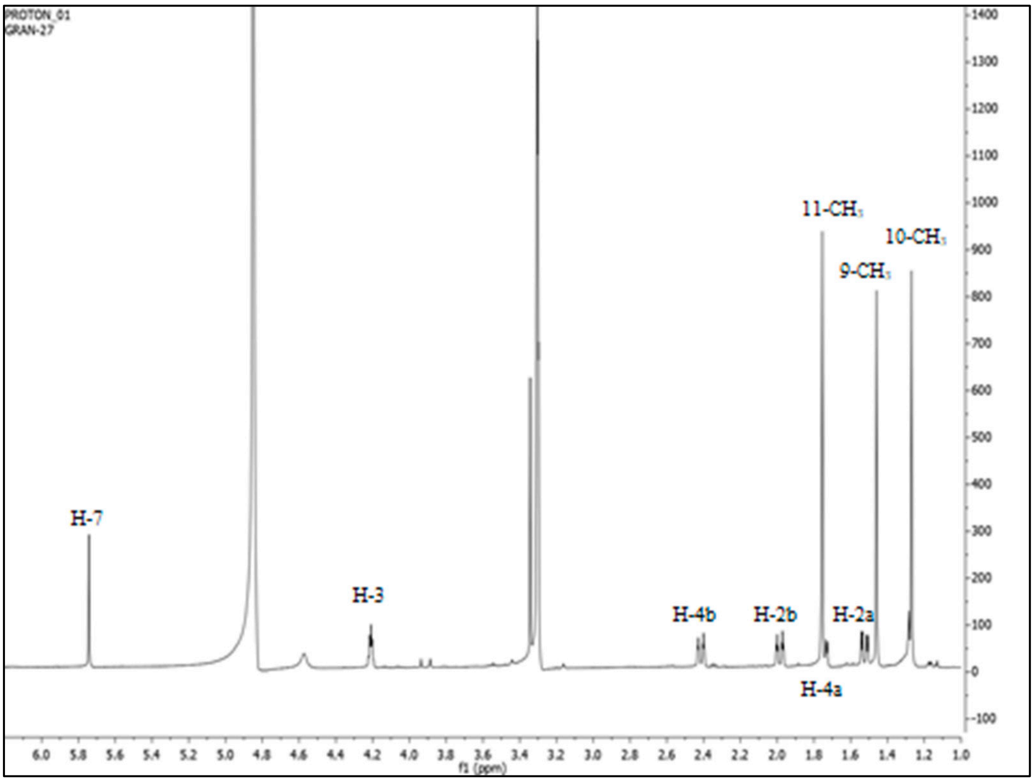

Figure S42. ^{1}H-NMR spectrum of compound **11** (CD_3OD, 500MHz).

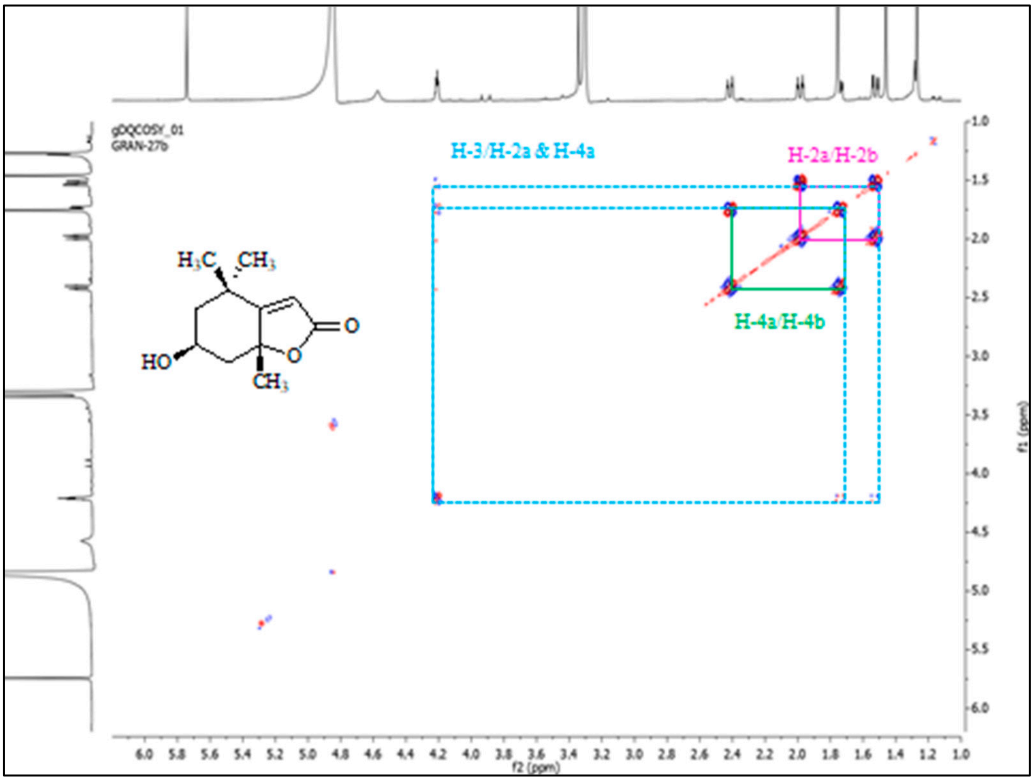

Figure S43. gDQCOSY spectrum of compound **11** (CD_3OD, 500MHz).

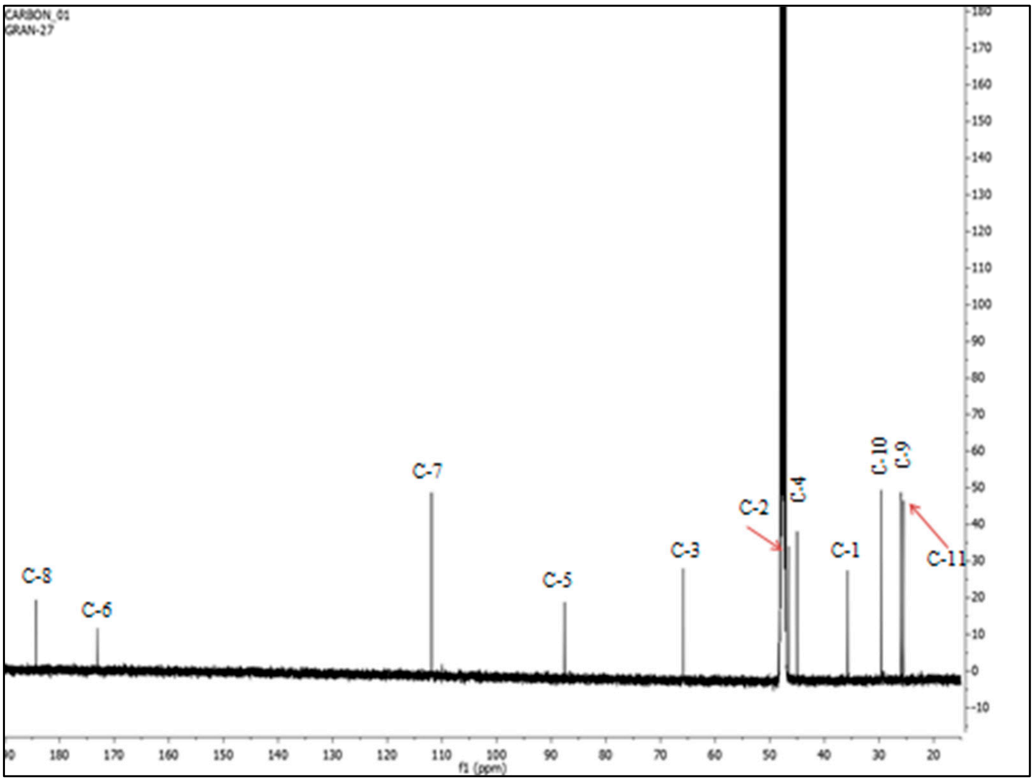

Figure S44. ^{13}C-NMR spectrum of compound **11** (CD_3OD, 125MHz).

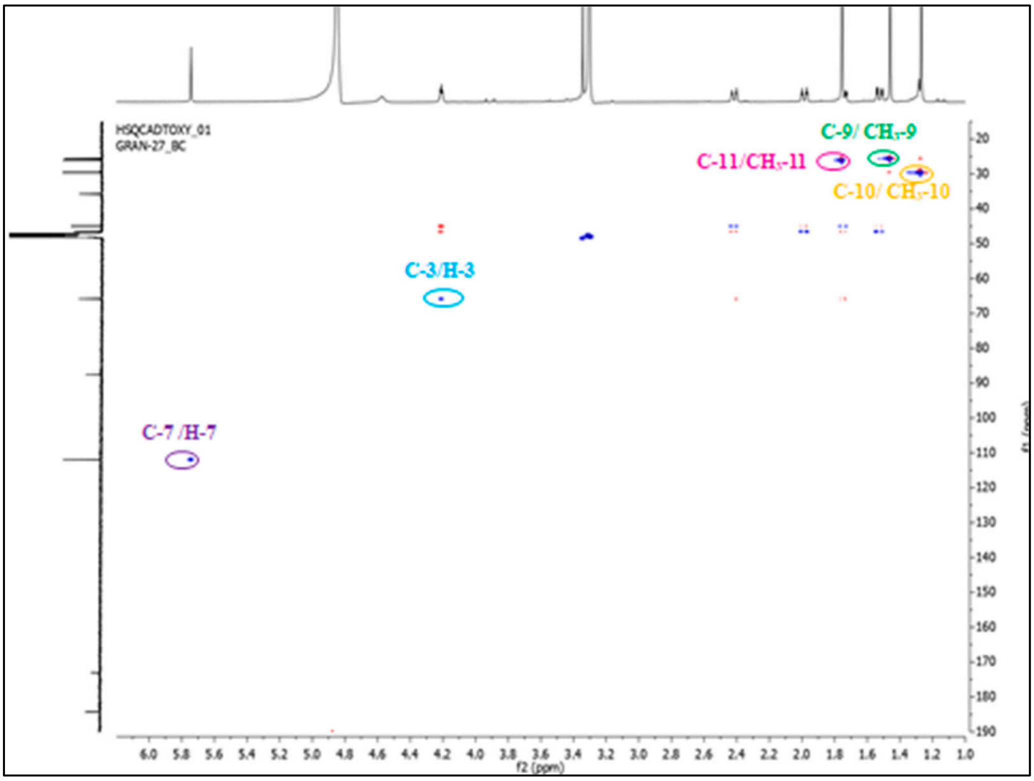

Figure S45. gHSQCAD spectrum of compound **11** (CD_3OD, 500MHz).

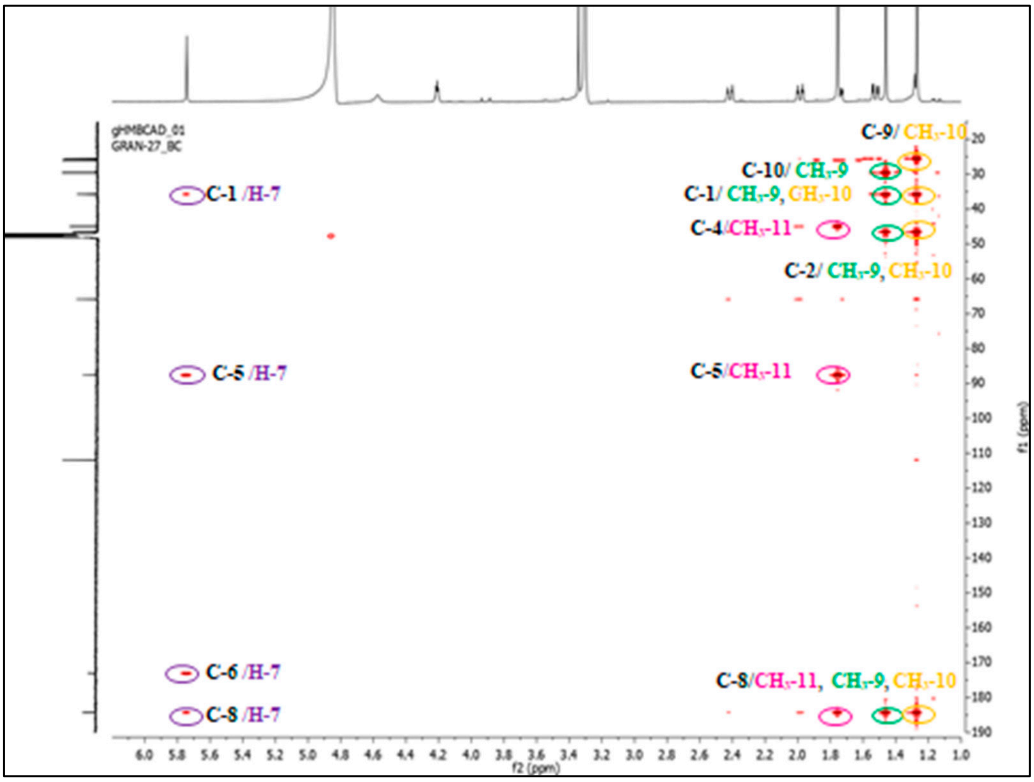

Figure S46. gHMBCAD spectrum of compound **11** (CD_3OD, 500MHz).

Table S12. ^{1}H NMR of compound **12** (CD_3OD, 500 MHz).

No	δ_H	H	J (Hz)
1	-	-	-
2	-	-	-
3	6.46	1	s
4	-	-	-
5	-	-	-
6	6.05	1	d (J = 2.0)
7	-	-	-
8	6.26	1	d (J = 2.0)
9	-	-	-
10	-	-	-
1′	-	-	-
2′	7.80	1	d (J = 8.5)
3′	6.88	1	d (J = 8.5)
4′	-	-	-
5′	6.88	1	d (J = 8.5)
6′	7.80	1	d (J = 8.5)

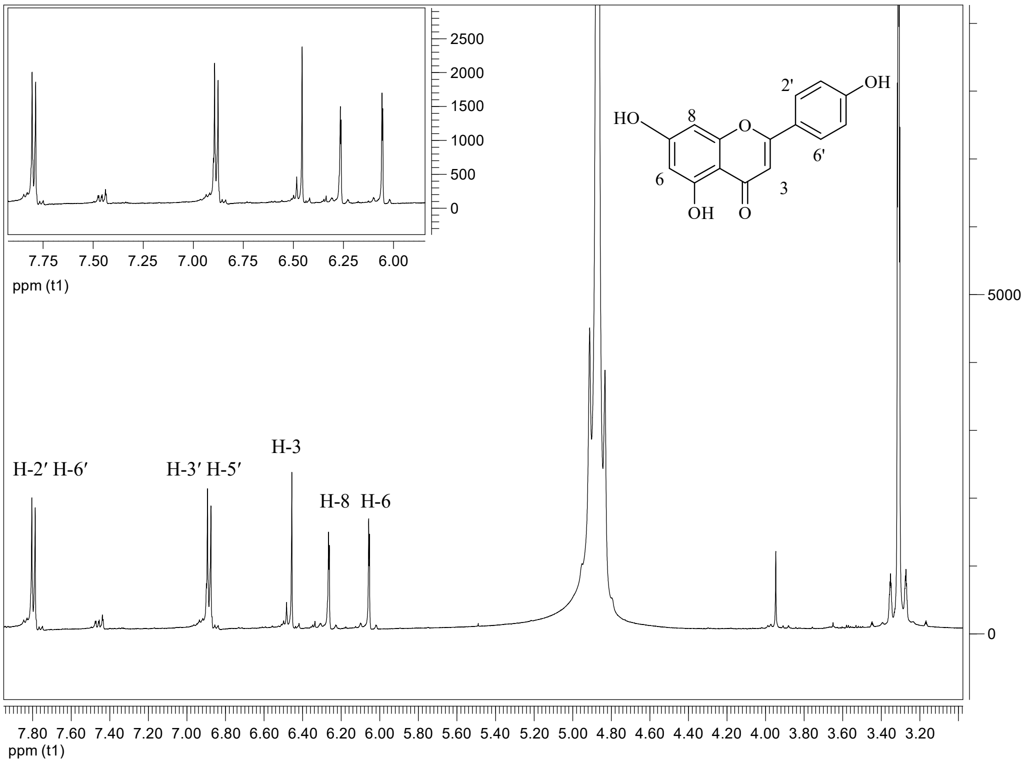

Figure S47. ^{1}H-NMR spectrum of compound **12** (CD_3OD, 500MHz).

Table S13. ^{1}H NMR of compound **13** (CD_3OD, 500 MHz).

No	δ_H	H	J (Hz)
1	-	-	-
2	-	-	-
3	6.51	1	s
4	-	-	-
5	-	-	-
6	6.19	1	s
7	-	-	-
8	6.42	1	s
9	-	-	-
10	-	-	-
1′	-	-	-
2′	7.37	1	o.s
3′	-	-	
4′	-	-	-
5′	6.90	1	d (J= 8.5)
6′	7.36	1	o.s

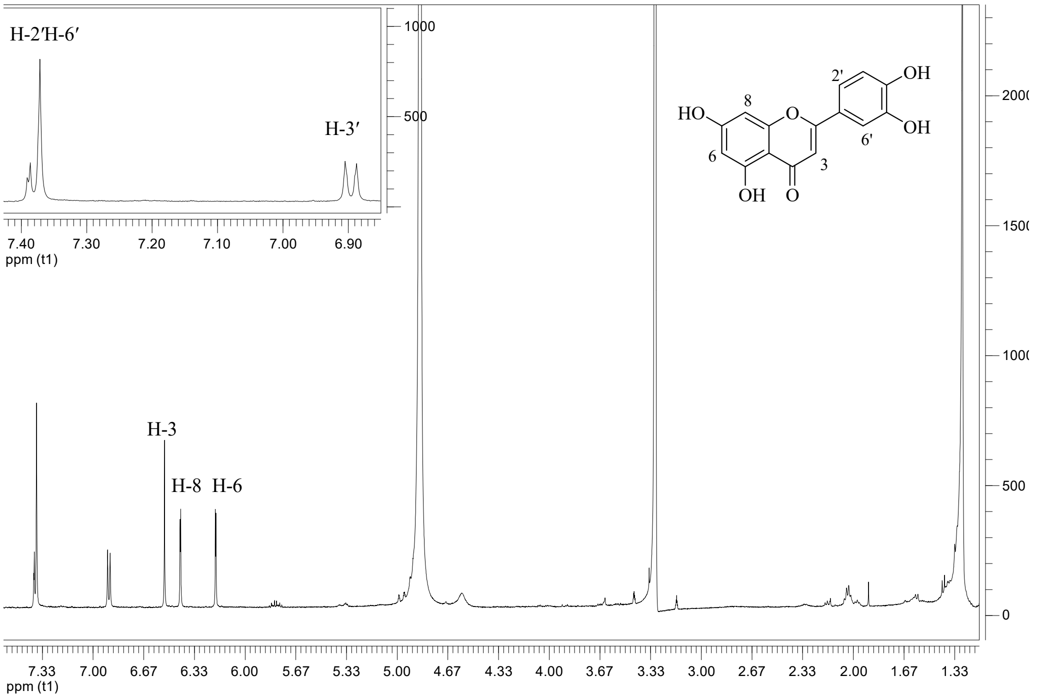

Figure S48. ^{1}H-NMR spectrum of compound **13** (CD_3OD, 500MHz).

Table S14. ^{1}H NMR of compound **14** (CD_3OD, 500 MHz).

No	δH	H	J (Hz)
1	-	-	-
2	-	-	-
3	-	-	-
4	-	-	-
5	-	-	-
6	-	-	-
7	-	-	-
8	6.47	1	s
9	-	-	-
10	-	-	-
1′	-	-	-
2′	7.63	1	br s
3′	-	-	-
4′	-	-	-
5′	6.92	1	d (J = 14.0)
6′	7.55	1	d (J = 14.0)
-OCH_3	3.90	3	s
-OCH_3	3.80	3	s

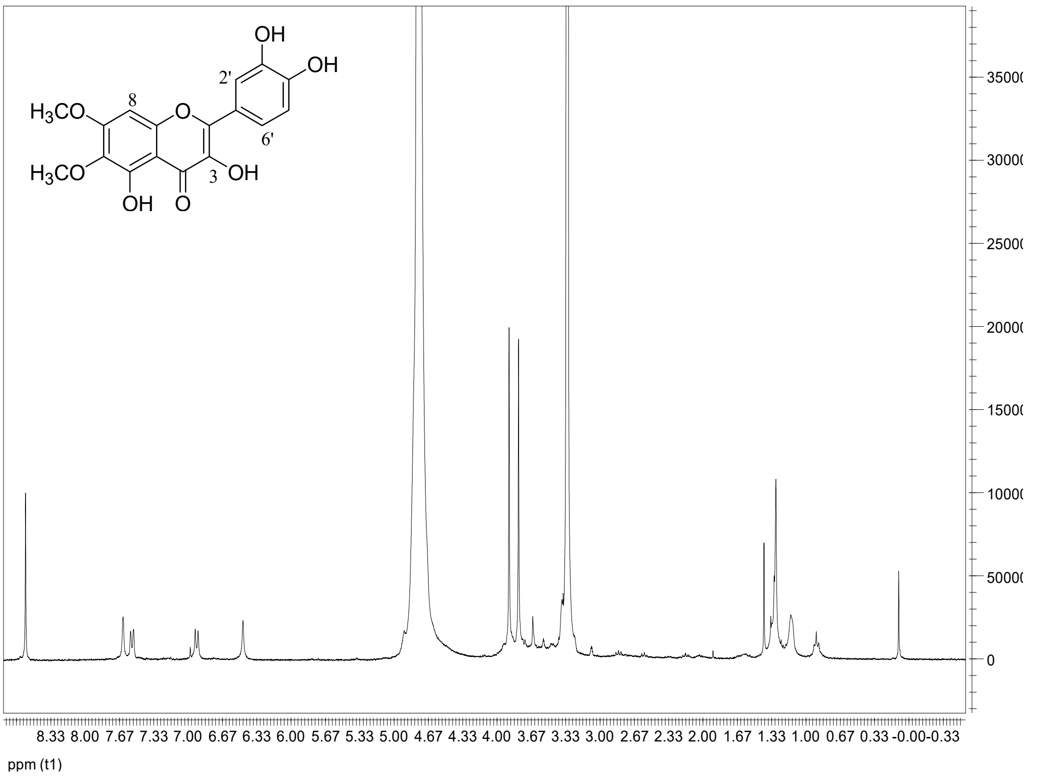

Figure S49. ^{1}H-NMR spectrum of compound **14** (CD_3OD, 500MHz).

Table S15. ^{1}H NMR of compound **15** (CD_3OD, 500 MHz).

No	δH	H	J (Hz)
1	-	-	-
2	-	-	-
3	6.66	1	s
4	-	-	-
5	-	-	-
6	6.50	1	s
7	-	-	-
8	6.82	1	s
9	-	-	-
10	-	-	-
1′	-	-	-
2′	7.89	1	d (J = 8.5)
3′	6.93	1	d (J = 8.5)
4′	-	-	-
5′	6.93	1	d (J = 8.5)
6′	7.89	1	d (J = 8.5)
1″	5.07	1	d (J = 8.5)
2″	3.48	1	o.s
3″	o.s	1	o.s
4″	o.s	1	o.s
5″	3.53	1	o.s
6″a	3.72	1	dd (J = 12.5, 5.5)
6″b	3.54	1	dd (J = 12.5, 7.0)

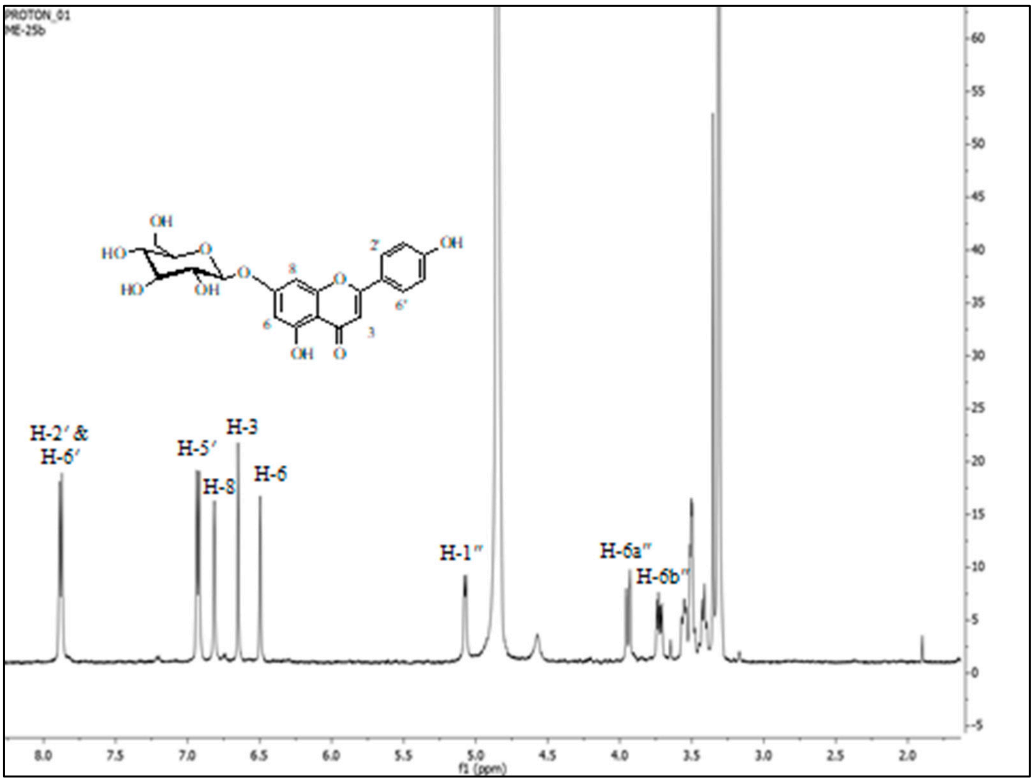

Figure S50. ^{1}H-NMR spectrum of compound **15** (CD_3OD, 500MHz).

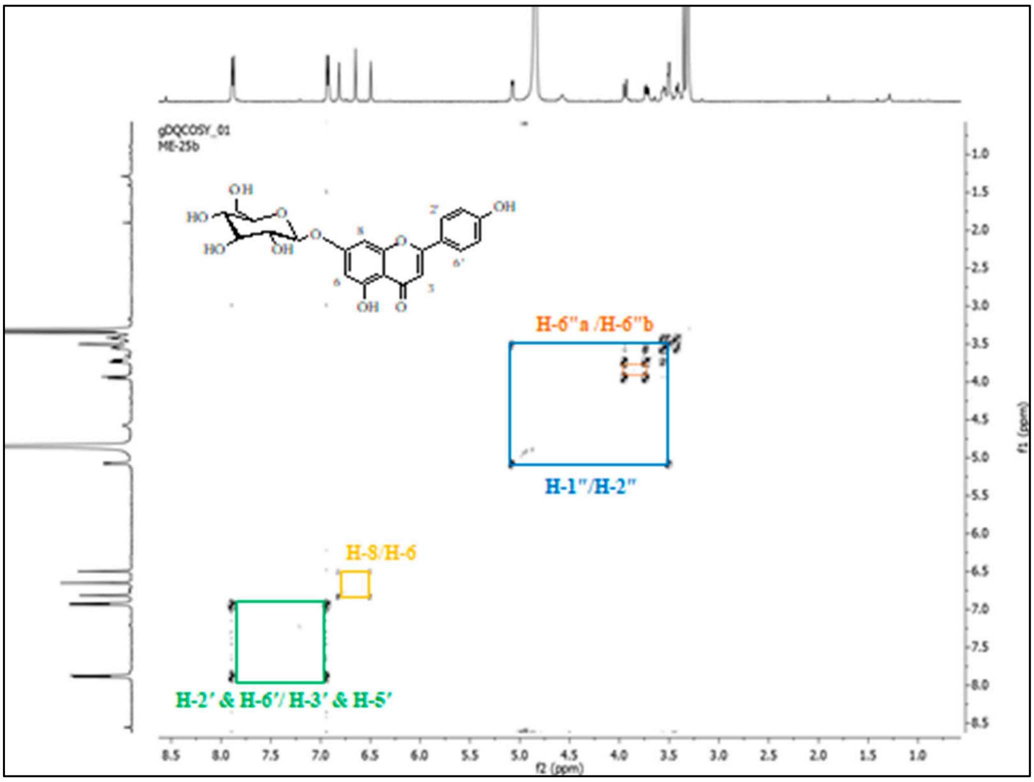

Figure S51. gDQCOSY spectrum of compound **15** (CD_3OD, 500MHz).

Table S16. ^{1}H NMR of compound **16** (CD_3OD, 500 MHz).

No	δ_H	H	J (Hz)
1	-	-	-
2	-	-	-
3	6.77	1	s
4	-	-	-
5	-	-	-
6	6.48	1	s
7	-	-	-
8	6.57	1	s
9	-	-	-
10	-	-	-
1′	-	-	-
2′	7.40	1	o.s
3′	-	-	
4′	-	-	-
5′	6.88	1	d (J = 8.5)
6′	7.38	1	o.s
1″	5.06	1	d (J = 7.0)
2″	3.16–3.64	1	o.s
3″	3.16–3.64	1	o.s
4″	3.16–3.64	1	o.s
5″	3.16–3.64	1	o.s
6″a	3.93	1	dd (J = 12.5, 1.5)
6″b	3.72	1	dd (J = 12.5, 5.5)

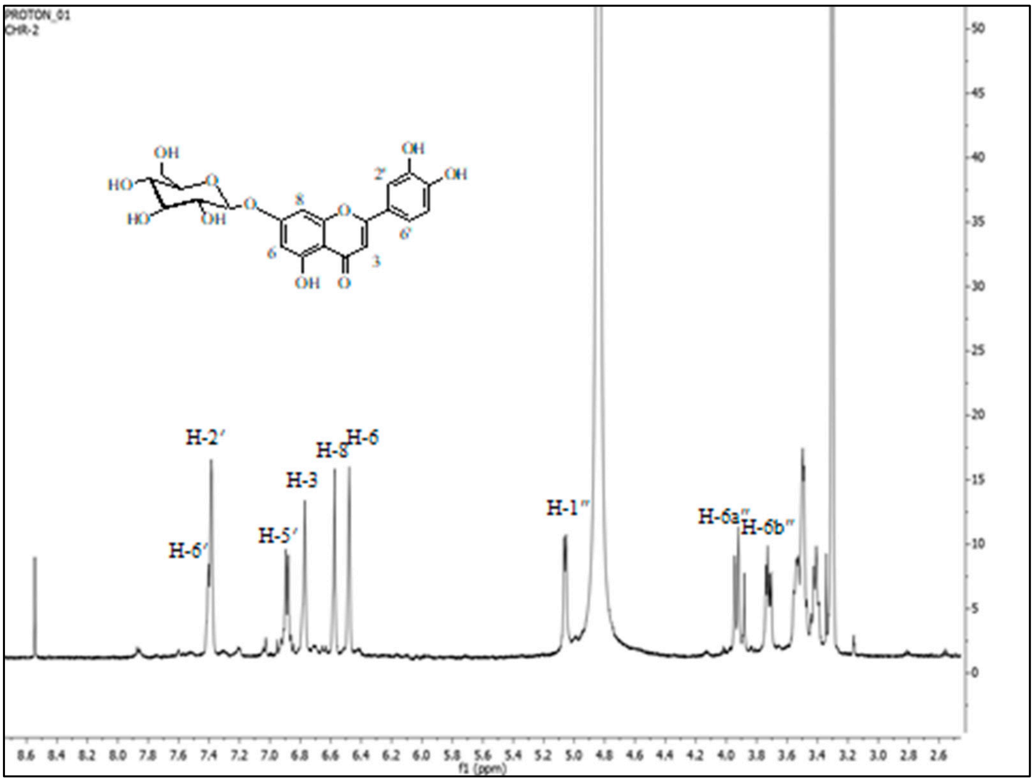

Figure S52. ^{1}H-NMR spectrum of compound **16** (CD_3OD, 500MHz).
